# MicroRNA Expression Profile during Aphid Feeding in Chrysanthemum (*Chrysanthemum morifolium*)

**DOI:** 10.1371/journal.pone.0143720

**Published:** 2015-12-09

**Authors:** Xiaolong Xia, Yafeng Shao, Jiafu Jiang, Xinping Du, Liping Sheng, Fadi Chen, Weimin Fang, Zhiyong Guan, Sumei Chen

**Affiliations:** College of Horticulture, Nanjing Agricultural University, Nanjing, China; Kunming University of Science and Technology, CHINA

## Abstract

MicroRNAs (miRNAs) are important regulators of gene expression, affecting many biological processes. As yet, their roles in the response of chrysanthemum to aphid feeding have not been explored. Here, the identity and abundance of miRNAs induced by aphid infestation have been obtained using high-throughput Illumina sequencing platform. Three leaf small RNA libraries were generated, one from plants infested with the aphid *Macrosiphoniella sanbourni* (library A), one from plants with mock puncture treatment (library M), and the third from untreated control plants (library CK). A total of 7,944,797, 7,605,251 and 9,244,002 clean unique reads, ranging from 18 to 30 nucleotides (nt) in length, were obtained from library CK, A and M, respectively. As a result, 303 conserved miRNAs belonging to 276 miRNAs families and 234 potential novel miRNAs were detected in chrysanthemum leaf, out of which 80, 100 and 79 significantly differentially expressed miRNAs were identified in the comparison of CK-VS-A, CK-VS-M and M-VS-A, respectively. Several of the differentially abundant miRNAs (in particular miR159a, miR160a, miR393a) may be associated with the plant's response to aphid infestation.

## Introduction

Small RNAs (smRNAs), ranging in length from 18 to 40 nucleotides (nt), regulate gene expression via their interaction with specific mRNA targets, thereby playing a significant role throughout plant growth and development [1, 2]. MicroRNAs (miRNAs), approximately 22 nt in size, are the most abundant species of endogenous noncoding smRNAs in plants [3, 4]. They have been implicated in the response to both biotic and abiotic stress [5, 6], by guiding the cleavage or repressing the translation of their target mRNAs, which is based on nearly perfect complementarity with the targets, resulting in gene silencing at post-transcriptional levels [3, 7].

The detection of miRNAs brings us into a new vision in the comprehension of gene regulation in plants. Evidence from the discovery that miR398 targeting two Cu/Zn superoxide dismutases (*CSD1* and *CSD2*) and a copper chaperone for *CSD1* responds to both oxidative stress and copper deficiency, indicating that miRNAs participate in plant stress responses [8, 9]. Besides abiotic stresses, miRNAs also play roles in different types of biotic stress. In *Arabidopsis thaliana*, miR393, which was induced by bacterial peptide flg22 treatment, repressed the expression of the transport inhibitor responsive 1 (*TIR1*) gene involved in auxin signaling pathway [10]. While miR160, miR167 and miR393 were all up-regulated, miR825 was down-regulated in *A*. *thaliana* leaves infected with certain bacterial pathogens [11]. In *A*. *thaliana*, *Brassica rapa* and tobacco plants inoculated with a pathogenic virus, various miRNAs are up-regulated (miR156 and miR164 in *A*. *thaliana* [12], miR156, miR160 and miR164 in tobacco [13], and miR158 and miR1885 in *B*. *rapa* were induced [14]). Meanwhile, Lu *et al*. [15] showed that the abundance of 10 out of 11 miRNA families was reduced in pine galled stem infected by endemic rust fungus.

In addition to bacterium, fungus and virus, the suggestion that smRNAs can also regulate the plant-herbivore interactions arises from the observed suppression of certain *Nicotiana attenuata* genes active in jasmonate and ethylene signaling, due to the silencing of genes encoding RNA-dependent RNA polymerase (RdRp) or Dicer-like proteins (DCLs), both of which are key components of smRNA biosynthesis [16–18]. Kettles *et al*. [19] indicated that the resistance of *A*. *thaliana* to green peach aphid (*Myzus persicae*) is regulated by miRNAs. In melon, 23 conserved miRNAs families and 18 cucurbit-specific miRNAs were identified after aphid infestation [4]. And the comparative analysis showed that the majority of conserved miRNA families were induced during the early stages of aphid feeding in the resistant plants, which was opposite to the susceptible one. The interaction of *N*. *attenuata* and *Manduca sexta* identified 59 herbivore-responsive miRNAs and 2 *trans*-acting small interfering RNAs (tasiRNA) targeted by miRNAs [20]. Further experiments revealed that these miRNAs and tasiRNAs responded specifically to either mechanical wounding or wounding plus oral secretions from *M*. *sexta* larvae, and were acted in JA-dependent or -independent ways. Some miRNAs performed similarly to aphid feeding across species, such as miR164, miR167, miR390 and miR393, whose expression were induced in both Arabidopsis and resistant plant of melon. However, there were also some contrary examples, such as miR162, which was up-regulated in melon but down-regulated in *A*. *thaliana*, suggesting the specificity of miRNAs expression profiles in response to aphid treatment between different species [4, 21].

Chrysanthemum (*Chrysanthemum morifolium* Ramat.) is a popular ornamental species worldwide, and especially in China [22, 23]. It is easily attacked by aphids from seedling to flowering. *Macrosiphoniella sanbourni*, a serious insect of chrysanthemum, not only interferes with vegetative growth, but also compromises flower quality and yield. The purpose of the present study was to document which miRNAs are associated with the plant's response to aphid infestation, and to characterize their abundance during an episode of aphid infestation. In order to figure out the potential impacts of aphid stylets, a mock puncture treatment is designed to partially simulate the mechanical stress resulting from aphid penetration. This work would lay a foundation for future researches on miRNAs regulating resistance to aphid in chrysanthemum.

## Results

### An overview of small RNA (smRNA) libraries sequencing

**Fig 1:**
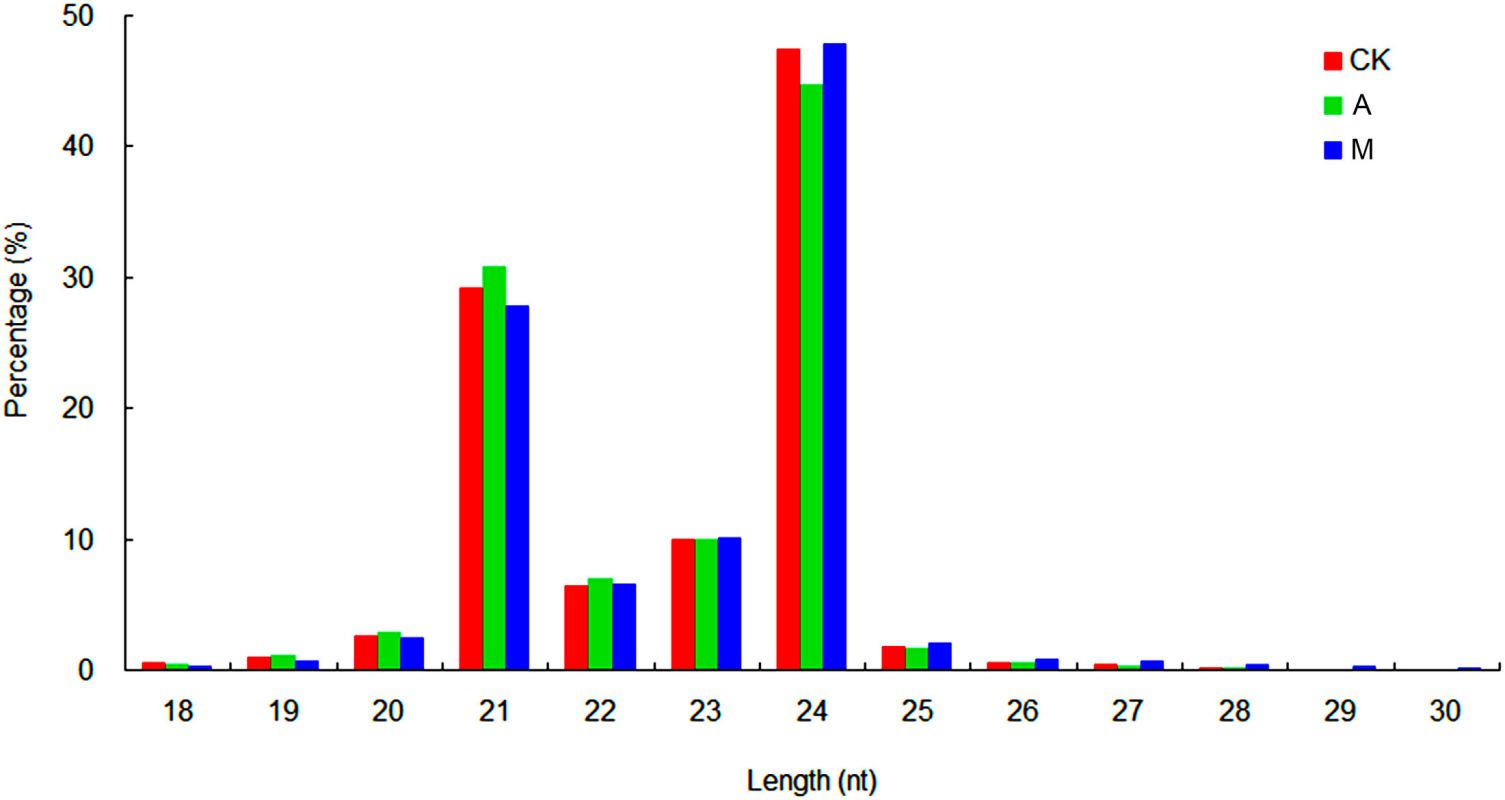
Size distribution of small RNA sequences in library CK, A and M. CK: control; A: aphid infestation treatment; M: mock puncture treatment; nt: nucleotide.

Three smRNA libraries were constructed from RNA extracted from pooled leaf material taken from chrysanthemum cultivar ‘Nannongxunzhang’ plants (aphid resistant) at various time points. The three treatments were: (A) aphid feeding treatment, (CK) control and (M) mock puncture treatment. About 22.1 million raw reads were produced in library CK, and approximately 21.8 and 26.8 million reads in library A and M, independently. After removal of low quality reads and those harboring adaptor sequence, a total of 7,944,797, 7,605,251 and 9,244,002 clean unique reads, ranging from 18 to 30 nucleotides (nt) in length, were obtained from library CK, A and M, respectively (Table 1). All clean reads were deposited in the NCBI Sequence Read Archive database (http://trace.ncbi.nlm.nih.gov/Traces/sra_sub/sub.cgi?) under accession number SRP051318. (Accession number SRS796738 for library CK; Accession number SRS799803 for library A; Accession number SRS799804 for library M). The proportion of clean reads was >99.49% in each library. As seen in Fig 1, the most abundant length was 24 nt, just as for rice, *Arabidopsis* and soybean. The three libraries each harbored a similar proportion of miRNAs (about 0.281% of unique reads and 5.021% of the total reads), rRNA (1.437% and 7.765%), snRNA (0.014% and 0.009%), snoRNA (0.005% and 0.004%) and tRNA (0.175% and 1.516%). The remaining unannotated reads were used to identify the new miRNAs.

**Table 1:** An overview of small RNA categories.

Category	Unique (CK)	Total (CK)	Unique (A)	Total (A)	Unique (M)	Total (M)
Raw reads		22,125,323		21,796,851		26,837,689
Clean reads	7,944,797 (100%)	21,931,156 (100%)	7,605,251 (100%)	21,586,815 (100%)	9,244,002 (100%)	26,633,282 (100%)
miRNA	22,898 (0.29%)	1,101,293 (5.02%)	21,525 (0.28%)	1,121,036 (5.19%)	25,202 (0.27%)	1,291,155 (4.85%)
rRNA	109,357 (1.38%)	1,507,036 (6.87%)	110,623 (1.46%)	1,727,120 (8.00%)	136,907 (1.48%)	2,243,391 (8.42%)
snRNA	1,116 (0.014%)	1,964 (0.009%)	1,169 (0.015%)	2,047 (0.009%)	1,252 (0.014%)	2,283 (0.009%)
snoRNA	393 (0.005%)	833 (0.004%)	432 (0.006%)	935 (0.004%)	483 (0.005%)	1,064 (0.004%)
tRNA	12,881 (0.16%)	292,946 (1.34%)	13,422 (0.18%)	366,519 (1.70%)	17,176 (0.19%)	403,227 (1.51%)
unannotated	7,798,152 (98.15%)	19,027,084 (86.76%)	7,458,080 (98.07%)	18,369,158 (85.09%)	9,062,982 (98.04%)	22,692,162 (85.20%)

CK: control; A: aphid infestation treatment; M: mock puncture treatment.

### Identification of conserved miRNAs and their expression profiles

**Fig 2:**
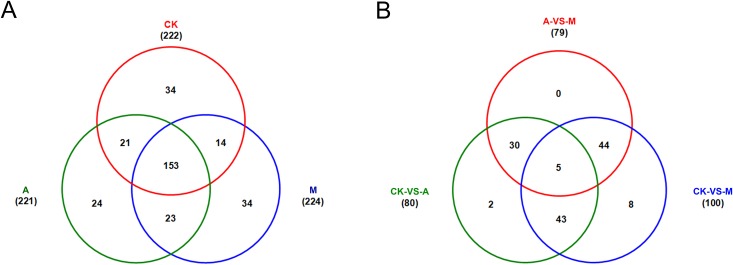
The expression profiles of conserved microRNAs (miRNAs). CK: control; A: aphid infestation treatment; M: mock puncture treatment. A: number of miRNAs detected in library CK, A and M; B: number of differentially expressed miRNAs identified in CK-VS-A, CK-VS-M and M-VS-A comparisons.

By matched to known miRNAs from miRBase database, a total of 303 conserved miRNAs belonging to 276 miRNAs families were identified in the three libraries (S1 Table). Of these, 222 conserved miRNAs were detected in library CK, and 221 and 224 miRNAs in library A and M, respectively. Among them, 34, 24 and 34 miRNAs were specifically expressed in library CK, A and M, respectively; 174, 176 and 167 miRNAs were co-expressed in library CK and A, library A and M or library CK and M, respectively; and 153 miRNAs were simultaneously expressed in library CK, A and M (Fig 2A, S1 Table).

**Fig 3:**
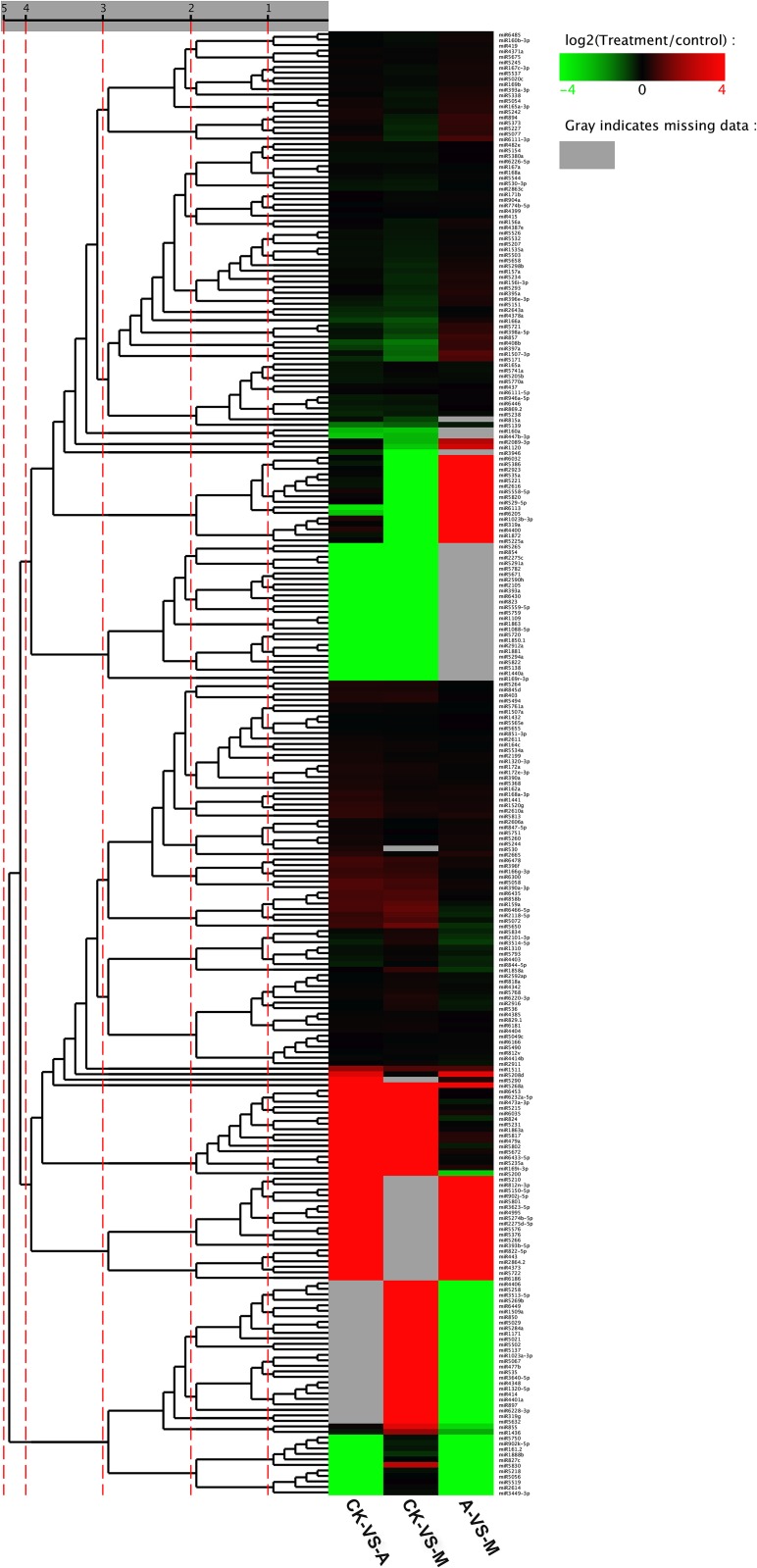
Heat map of differential expression and cluster analysis of conserved microRNAs (miRNAs). MiRNAs who have similar pattern of differential expression in different library comparisons are clustered together. Red: up-regulation; green: down-regulation; gray: miRNA has no expression in at least one library. CK: control; A: aphid infestation treatment; M: mock puncture treatment. CK-VS-A: comparison between library CK and A; CK-VS-M: comparison between library CK and M; M-VS-A: comparison between library M and A.

According to fold-change (|log_2_Ratio(treatment/control)| ≥ 1.0) and *P*-value (< 0.05), there were 80 significantly differentially expressed miRNAs (39 miRNAs up-regulated and 41 miRNAs down-regulated, 39/41) in library CK and A comparison (CK-VS-A), and 100 (49/51) and 79 (39/40) miRNAs in CK-VS-M and M-VS-A, respectively (S2 Table), of which 2 and 8 miRNAs were specifically expressed in CK-VS-A and CK-VS-M, respectively; 48, 49 and 35 miRNAs were co-expressed in CK-VS-A and CK-VS-M, CK-VS-M and M-VS-A or CK-VS-A and M-VS-A, respectively; and 5 miRNAs were simultaneously expressed in CK-VS-A, CK-VS-M and M-VS-A (Figs 2B and 3, S2 Table).

### Prediction of conserved miRNA targets and mapping of cleavage sites in the targets

**Fig 4:**
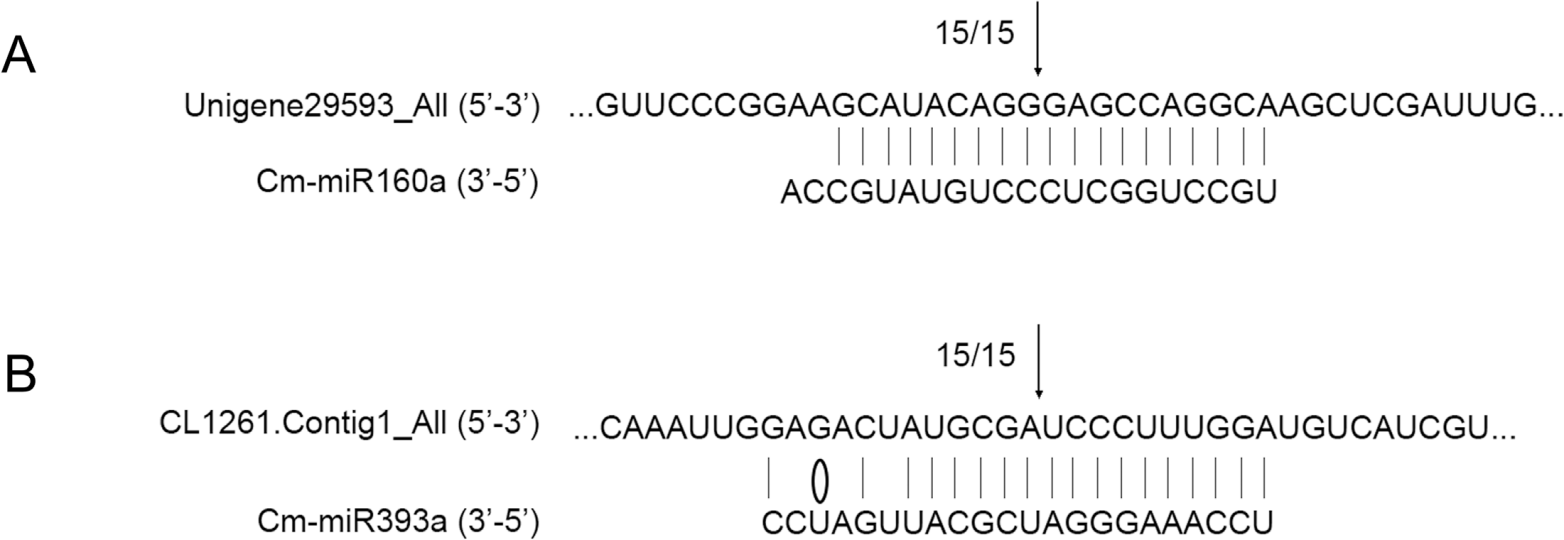
Target validation of miR160a and miR393a in chrysanthemum. A: cleavage site in the target of miR160a; B: cleavage site in the target of miR393a. The 5’ end of the cleavage product is indicated by arrow with the frequency of clones. Vertical dash: Watson-Crick pairing; Circle: G:U wobble pairing.

Based on the characteristic of near-perfect complementarity with their targets of plant miRNAs, the transcriptome of chrysanthemum was used to predict targets of the conserved miRNAs according to Allen *et al*. [24, 25]. In the comparison between CK and A (CK-VS-A), 26 out of 80 differentially expressed miRNAs were predicted to target 145 genes (26/80/145). And the corresponding numbers in CK-VS-M and M-VS-A were 38/100/159 and 27/79/110, respectively (S3 Table). For examining the influences of the conserved miRNAs on their predicted target genes, RNA ligase-mediated 5’ rapid amplification of cDNA ends (RLM-5’ RACE) was performed to map internal cleavage sites in the targets using a GeneRacer kit (Invitrogen, Carlsbad, CA). The miR160a-guided cleavage of Unigene29593_All which encodes an *ARF* gene and the miR393a-guided cleavage of CL1261.Contig1_All encoding one TRANSPORT INHIBITOR RESPONSE 1 (TIR1) protein were validated in chrysanthemum (Fig 4).

### Gene ontology (GO) functional classification

**Fig 5:**
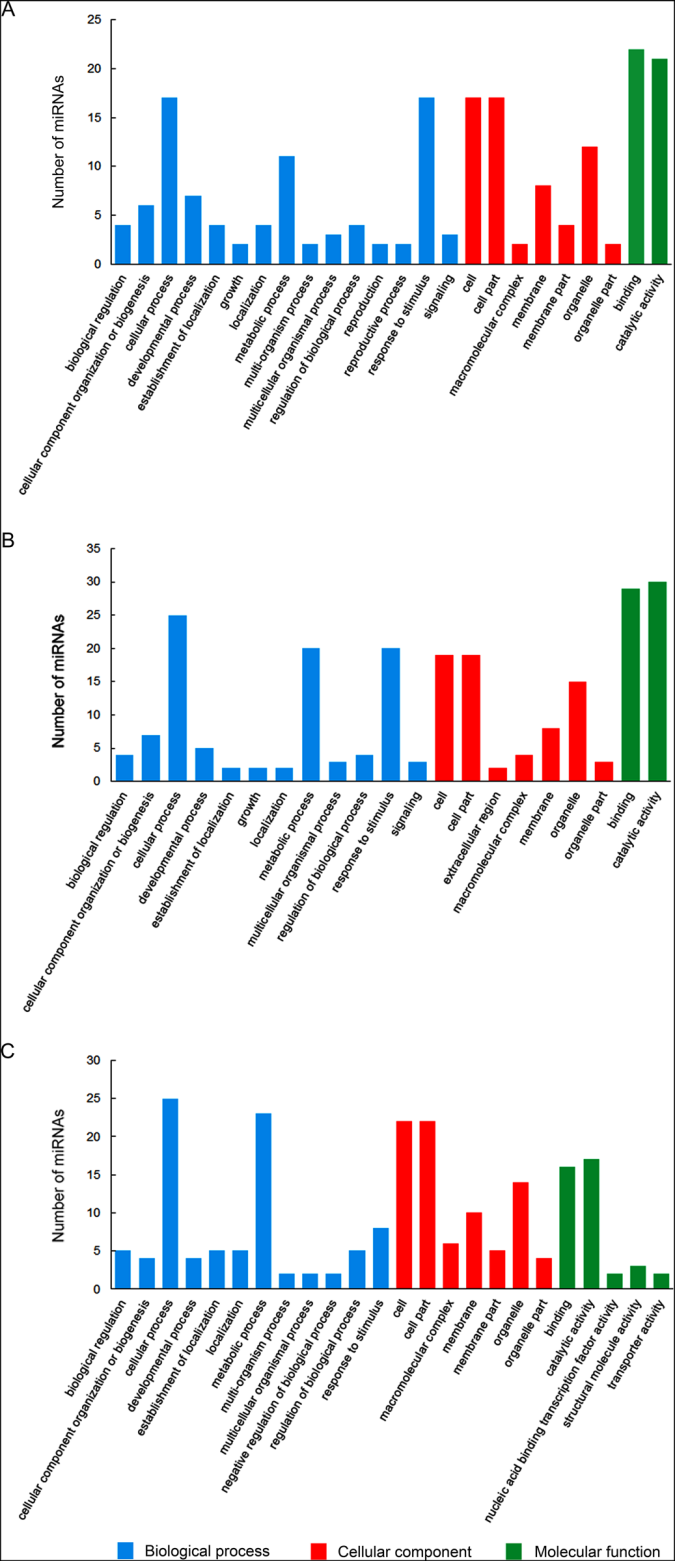
Gene Ontology (GO) functional classification of targets of conserved microRNAs (miRNAs). Targets were annotated in three categories: biological process (blue), cellular component (red) and molecular function (green). CK: control; A: aphid infestation treatment; M: mock puncture treatment. A: comparison between library CK and A (CK-VS-A); B: comparison between library CK and M (CK-VS-M); C: comparison between library M and A (M-VS-A).

To investigate the potential roles of miRNAs, we performed GO analysis in terms of the targets of conserved miRNAs that were differentially expressed. In CK-VS-A comparison, 88 target genes were categorized as “biological process”, 62 as “cellular component” and 43 as “molecular function” (88/62/43), and 97/70/59 and 90/83/40 in CK-VS-M and M-VS-A, respectively (Fig 5). As shown in Fig 5, most of target genes were categorized into “biological process”, and majority of them were associated with cellular process, metabolic process and response to stimulus in CK-VS-A, CK-VS-M and M-VS-A. Furthermore, in terms of cellular component, the majorities were involved in cell, cell part and organelle, and most of the targets were involved in binding and catalytic activity in terms of molecular function. Moreover, on the aspect of numbers of target genes in the categories, these genes were mainly enriched in binding and catalytic activity in CK-VS-A and CK-VS-M.

### Prediction of potential novel miRNAs

**Fig 6:**
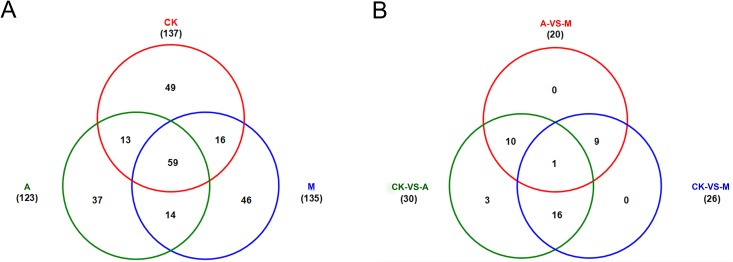
The expression profiles of potential novel microRNAs (miRNAs). CK: control; A: aphid infestation treatment; M: mock puncture treatment. A: number of miRNAs detected in library CK, A and M; B: number of differentially expressed miRNAs identified in CK-VS-A, CK-VS-M and M-VS-A comparisons.

In total, 234 potential novel miRNAs were identified in the three libraries (S4 Table). Of these, 137 potential novel miRNAs were detected in library CK, and 123 and 135 miRNAs in library A and M, respectively. Among them, 49, 37 and 46 miRNAs were specifically expressed in library CK, A and M, respectively; 72, 73 and 75 miRNAs were co-expressed in library CK and A, library A and M or library CK and M, respectively; and 59 miRNAs were simultaneously expressed in library CK, A and M (Fig 6A, S4 Table). The precursor sequences of these potential novel miRNAs were listed in S1 File.

According to fold-change (|log_2_Ratio(treatment/control)| ≥ 1.0) and *P*-value (< 0.05), there were 30 significantly differentially expressed miRNAs (11 miRNAs up-regulated and 19 miRNAs down-regulated, 11/19) in library CK and A comparison (CK-VS-A), and 26 (8/18) and 20 (11/9) miRNAs in CK-VS-M and M-VS-A, respectively (S5 Table), of which 3 potential novel miRNAs were specifically expressed in CK-VS-A comparison; 17, 10 and 11 genes were co-expressed in CK-VS-A and CK-VS-M, CK-VS-M and M-VS-A or CK-VS-A and M-VS-A, respectively; and 1 miRNAs were simultaneously expressed in CK-VS-A, CK-VS-M and M-VS-A (Fig 6B, S5 Table).

### miRNAs responding to aphid and mock puncture treatments

Of the 537 miRNAs, 153 conserved miRNAs and 59 potential novel miRNAs were found in all three treatments, occupying 39.5% of the total (Figs 2A and 6A). 24 conserved miRNAs and 37 potential novel miRNAs were specifically detected in plants subjected to aphid herbivory. There were 80 puncture-specific miRNAs, in which 34 miRNAs were conserved and 46 miRNAs were novel. 23 conserved miRNAs and 14 potential novel miRNAs were specific to both aphid and mock puncture treatments. Compared with the control (CK) (Figs 2B and 6B), 32 conserved miRNAs and 13 potential novel miRNAs were differentially responded to aphid feeding. 48 conserved miRNAs and 17 potential novel miRNAs differentially response to both aphid infestation and mock puncture were identified. Additionally, 52 conserved miRNAs and 9 potential novel miRNAs were differentially expressed during mock puncture treatment.

### Validation of the expression of miRNAs by stem-loop quantitative real-time PCR (qRT-PCR)

**Fig 7:**
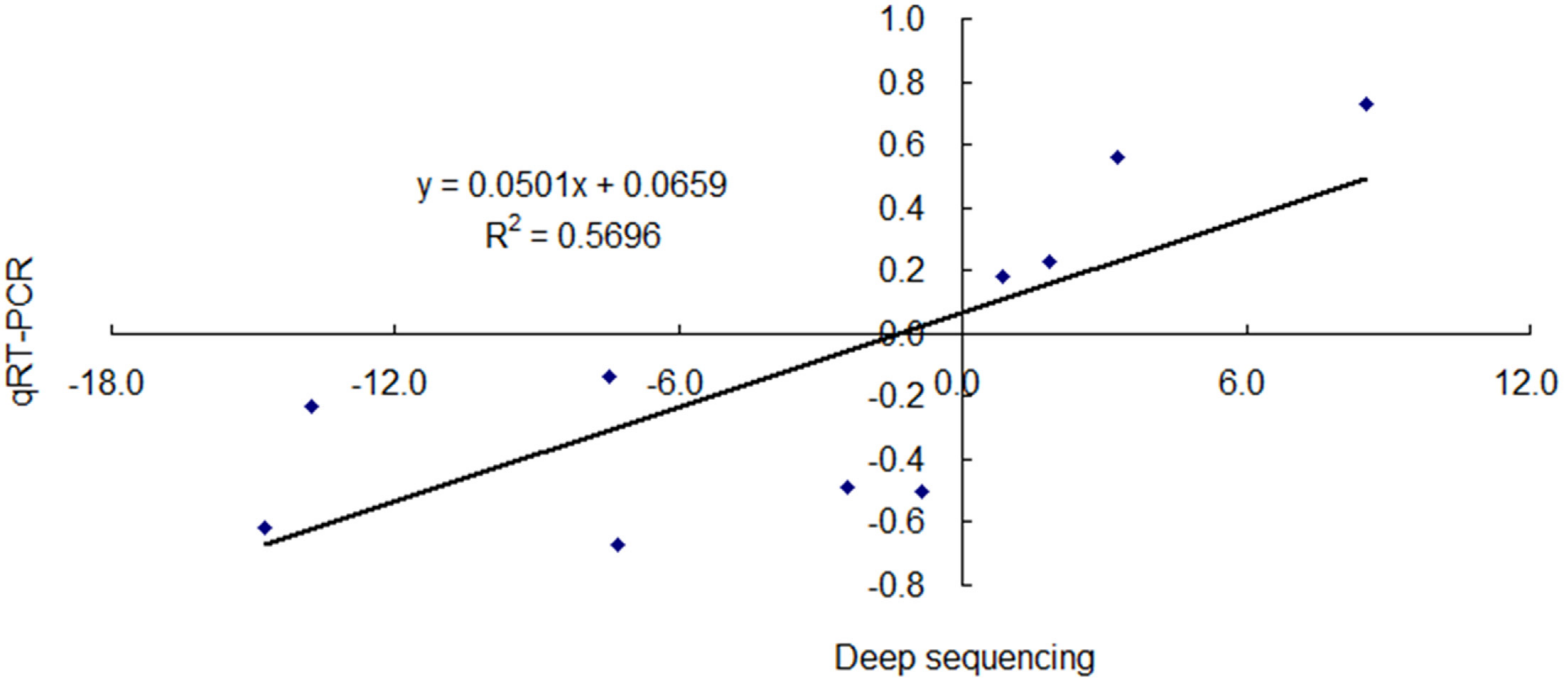
Validation of the expression of microRNAs (miRNAs) from deep sequencing in leaf tissues of chrysanthemum. Correlation of fold change was analyzed by deep sequencing (*x* axis) with data obtained using qRT-PCR (*y* axis).

To validate the results of Illumina deep sequencing, several miRNAs from library CK and A (CK: control; A: aphid infestation treatment) were chosen randomly for stem-loop qRT-PCR. For comparison of fold change between Illumina sequencing and qRT-PCR, scatterplots were generated using the log_2_ fold change determined by Illumina sequencing and qRT-PCR. As shown in Fig 7, the qRT-PCR results revealed that the expression of these miRNAs showed a similar tendency (*R* ^*2*^ = 0.57) [26] with the Illumina sequencing data, suggesting the reproducibility and accuracy of sequencing results.

## Discussion

As important regulators of gene expression, miRNAs are widely discovered in animals and plants. Thousands of miRNAs of plants were annotated in the miRBase database, including rice and Arabidopsis [27, 28]. Here, the capacity of the Illumina sequencing platform to generate large quantities of sequence in a single run has been exploited to achieve a global picture of the miRNA content of chrysanthemum, a larger genome polyploid, and in particular to identify those which may be involved in the plant's response to aphid infestation. As a result, a total of 303 conserved miRNAs belonging to 276 miRNAs families and 234 potential novel miRNAs were identified in three libraries constructed from pooled leaf tissues of *Chrysanthemum morifolium* ‘nannongxunzhang’ that were collected at different time points with (A) or without (CK) aphid infestations and mock puncture treatment (M) (S1 and S4 Tables).

In this study, the abundance of a large number of miRNAs was increased in response both to aphid feeding (CK-VS-A) and mock puncture treatments (CK-VS-M). To figure out the potential role of these miRNAs, several specific miRNAs that are candidates for the resistance to aphids were further analyzed. Chrysanthemum miR159a induced by aphid feeding and mock puncture treatments was predicted to target a *GAMYB-like 2* gene. The *GAMYB* or *GAMYB-like* genes encode R2R3 MYB domain transcription factors involving in gibberellin (GA) signaling pathway [29]. In *Arabidopsis*, miR159 suppresses the expression of *GAMYB-like* gene(s) regulating cell proliferation in vegetative tissues and promoting programmed cell death in the aleurone layer [30]. Pegadaraju and co-workers [31] reported that the feeding of the aphid *Myzus persicae* on *A*. *thaliana* up-regulated the expression of a senescence-associated gene (SAG), triggering the premature senescence of the aphid-infested leaves, thereby restricting the multiplication of the aphids, contributing to the defense against *M*. *persicae* in *A*. *thaliana*. Aphid feeding could create a strong sink in insect-infested tissue, leading to increased flow of nutrients to those tissues in the host [32]. So we propose that miR159a regulation might be implicated in the PCD induced by aphid infestation (aphid saliva and mechanical injury), which redirects the resource allocation and contributes to resist aphids.

Both miR160a and miR393a were down-regulated by both aphid infestation and the mock puncture treatment. The RLM 5’-RACE assay successfully identified their targets in chrysanthemum as members of Auxin Response Factors (ARFs) family and *TRANSPORT INHIBITOR RESPONSE 1* (*TIR1*) (Fig 4), respectively. The transcript abundance of the two targets was both increased in response to aphid feeding (S1 and S2 Figs). TIR1 is part of the ubiquitin-ligase complex SCF^TIR1^ mediating the degradation of Aux/IAA proteins that repress auxin signaling through heterodimerization with ARFs [33, 34]. Navarro *et al*. [10] showed that miR393 induced by a flagellin-derived peptide negatively regulated mRNAs for the F-box auxin receptors TIR1, AFB2 and AFB3, thereby repressing the auxin signaling and restricting *Pseudomonas syringae* growth, indicating auxin in the susceptibility of disease and miRNA-mediated suppression of auxin signaling in resistance. In different eco-types of *Arabidopsis*, Kuśnierczyk and co-workers [35] found that several genes which belong to the auxin biosynthesis pathway were up-regulated after green peach aphid (*M*. *persicae*) infestation. The induced down-regulation of miR160a and miR393a may suggest the involvement of miRNA-mediated gene regulation in the chrysanthemum-aphid interaction.

Unfortunately, among those conserved miRNAs, only half of them could be predicted to targets, of which two were further validated through RLM-5’ RACE method. The reasons that only a small number of validated targets may be as follows: the limited coverage of the chrysanthemum transcriptome; the limitation of the RLM-5’ RACE method which is hard to detect the low abundance of the cleavage products and is difficult to identify genes that are targeted by more than one miRNA; and in some cases, miRNAs might silence gene by repressing their translation rather than their transcription. Although there is a deal of conservation across species in terms of miRNA targets, it has been noted that some known miRNAs target additional, non-conserved genes in different species [4, 36]. An example is provided by miR159a, which in addition to *GAMYB-like 2*, also interacts with a gene encoding a 50S ribosomal protein. Similarly, miR397a targets a glucosidase gene in addition to *TIR1*. Degradome sequencing [37] and transient co-expression of miRNAs and their predicted targets in *Nicotiana benthamiana* leaves [38] both helps the prediction of target genes, which will be our ongoing study. Moreover, with the development of sequencing technologies, the completion and release of chrysanthemum genome and transcriptome sequence will be expectable and would aid in the identification of predicted conserved and additional target genes.

Furthermore, the mock puncture treatment was included in the present experiments in an attempt to separate the plant's wounding response caused by the insertion of the aphid stylet from its response to feeding. In spite of some differentiations between aphid stylet and puncture, for example, aphid stylets were often wrapped by saliva that contains a complicated mixture of enzymes and can lead to defense responses [39]. Also, the mechanical degree of puncture treatment should be different from aphid stylets. Results show that it does have some similarities between aphid feeding and puncture treatment. As seen in Figs 2B and 6B, 32 conserved miRNAs and 13 potential novel miRNAs were specifically differentially expressed in CK-VS-A; whereas 48 conserved miRNAs and 17 potential novel miRNAs were co-expressed in CK-VS-A and CK-VS-M, suggesting that miRNAs co-expressed in response to aphid feeding and puncture treatment might be involved in wound-induced responses by aphid, otherwise miRNAs may specifically respond to aphid sucking. These will allow us to find out the potential effects of aphid stylets and refine the processes of defense responses.

Here, we mainly focus on the discussions of conserved miRNAs. In the absence of whole genome annotation, the chrysanthemum transcriptome was also used to predict targets for potential novel miRNAs according to the criteria used by Allen *et al*. [24, 25]. However, the sequences of potential targets of miRNAs were too short to get reliable prediction.

Our results indicated that the interaction between chrysanthemum and *Macrosiphoniella sanbourni* shared some common with interactions between other plants and aphids in terms of changes in expression of miRNAs, such as miR159a, miR160a and miR393a which have been previously proposed to be involved in response to aphid attack in Arabidopsis and melon [4, 40], suggesting that present study could provide valuable clues for dissecting other plants’ responses to phloem feeding insects. We speculate that these miRNAs might be associated with the aphid infestation in chrysanthemum based on their expression profiles, and the further validations of the two targets of miR160a and miR393a in chrysanthemum suggested that these miRNAs are of worth to be studied further. Additional evidence should be collected to verify if these miRNAs contribute to the resistance of aphid in chrysanthemum, which will be our ongoing research.

## Conclusions

In summary, this study has demonstrated that both aphid infestation and mock puncture treatment induced a large change in the spectrum of miRNAs present in the chrysanthemum leaf. Some of the induced miRNAs are likely to be associated with the plant's defense response to aphid feeding, and thus are of interest in efforts to genetically manipulate aphid resistance in chrysanthemum. The number of differentially abundant miRNAs uncovered implies that multiple defense pathways are likely activated by aphid infestation, allowing the plant a degree of genetic flexibility in combating this form of biotic stress.

## Materials and Methods

### Plants growth

Cuttings of the aphid resistant *Chrysanthemum morifolium* cultivar ‘Nannongxunzhang’ were obtained from the Chrysanthemum Germplasm Resource Preserving Centre, Nanjing Agricultural University, China. Seedlings were grown in a 2:1 mixture of garden soil and vermiculite without added fertilizer under 80% relative humidity, a 16 h photoperiod (160 μmol m^-2^ s^-1^ photon flux density), and a day/night temperature of 25/18°C in a greenhouse. Uniform plants at the 6–8 leaf stage were used in all experiments.

### Aphid infestation and acupuncture treatment

Aphids (*Macrosiphoniella sanbourni* Gillette) were collected from field-grown chrysanthemum plants and second instar nymphs were raised for the infestation experiment (A). Because the aphids were from our own chrysanthemum planting base in ‘Suoshi’ village of nanjing in China, no specific permissions were required for any locations/activities. Aphid infestation (A) and mock puncture treatment (M) were carried according to Xia *et al*. [41]. For aphid infestation treatment (A), a set of 20 aphids was placed on the third fully expanded leaf (counting from the top of the stem), which was then enclosed by transparent, ventilated plastic cylinder (2 cm height × 5 cm diameter) and sealed at the base of the petiole. The same enclosure was also used for the control (CK) and mock puncture treatment (M). For the mock puncture treatment (M), designed to partially simulate the mechanical stress resulting from aphid penetration, the third fully expanded leaf of each plant was punctured 5 times at 0 h, 10 times at 24 h, and 15 times at 48 h with a needle (approximately 0.30 mm diameter) [42]. Leaves of three individual seedlings for each treatment were harvested at 0 h, 3 h, 6 h, 12 h, 24 h and 48 h. Before harvest, aphids were removed by spraying with 1% (v/v) SDS solution, which caused aphids to remove their mouthparts from plant tissues and then removed the aphids from the leaves by flushing the plants with deionized water. Harvested materials were immediately frozen in liquid nitrogen and stored at -80°C for the following experiments. The samples collected at defined time points of each treatment were pooled for sequencing.

### RNA extraction, small RNA library construction and Illumina deep sequencing

Total RNA from leaf tissue of three separate libraries (CK, M, A) was extracted using RNAiso reagent (TaKaRa, Otsu, Japan), following the manufacturer’s instructions. The integrity and quality of the total RNA was evaluated using a 2100 bioanalyzer RNA Nano chip device (Agilent, Santa Clara, CA, USA) and agarose gel electrophoresis, and the concentration was measured with a ND -1000 spectrophotometer (NanoDrop, Wilmington, DE).

After the total RNA isolation, low molecular weight (LMW) RNAs (18–30 nt) were isolated by polyacrylamide gel electrophoresis. The LMW RNAs were then ligated to a 5’ RNA adapter and a 3’ RNA adapter. A reverse transcription reaction followed by low cycle PCR amplification (initial denaturation at 98°C for 30 s, followed by 15 cycles of 98°C for 10 s, 60°C for 30 s and 72°C for 15 s, and then 72°C for 10 min) was performed to generate sufficient template for SBS-sequencing by synthesis, which can decrease the loss of nucleotides caused by the secondary structure. PCR products approximately 100 bp were collected by gel purification and sequenced on Illumina Genome Analyzer II platform according to manufacturer’s instructions (Illumina Inc, USA).

### Bioinformatic analysis of sequencing data

The raw reads from Illumina Genome Analyzer II were initially processed to remove adaptor sequences, low-quality reads as well as contaminants to get clean reads. Reads ranging from 18–25 nt in length were matched to known miRNAs from miRBase database (http://www.mirbase.org/) to identify conserved miRNA. To remove reads from ncRNA (noncoding RNA), such as rRNAs (ribosomal RNAs), tRNAs (transfer RNAs), snRNAs (small nuclear RNAs), snoRNAs (small nucleolar RNAs) and repeat RNA, we aligned the reads with Rfam (http://www.sanger.ac.uk/) and Genbank database (http://www.ncbi.nlm.nih.gov/). Sequences matching exons and introns of mRNA were also removed to avoid mRNA contamination using overlap software developed by BGI TechSolutions (Shenzhen, China). The remaining reads without any annotation were used to predict potential novel miRNAs by Mireap (http://sourceforge.net/projects/mireap) according to Friedländer *et al*. [43]. And because of the lack of genome sequence, EST and transcriptome sequences were used to find potential novel miRNAs in chrysanthemum. In the absence of whole genome annotation, the transcriptome of chrysanthemum was used to predict targets of the identified miRNAs according to the criteria used by Allen *et al*. [24, 25]. All sequencing data have been deposited at the sequence read archive (SRA) of NCBI.

The expression abundance of each miRNA was estimated by std, normalized expression level of miRNA in a sample. Normalization formula: Normalized expression = (Original counts of miRNAs / Total count of clean reads) * 10^6^. If the original miRNA expression in a library was zero, the normalized expression was adjusted to 0.01; if the miRNA gene expression of the two samples was less than 1, it was not used to analyze the differential expression due to its low expression. The fold change between two samples was calculated as: fold change = log_2_(treatment/control). The comparisons of the miRNA expression levels between the three libraries were performed using Poisson distribution (*P* < 0.05). *P*-value formula:

$$p(x/y)={\left(\frac{N_{2}}{N_{1}}\right)}^{y}\frac{(x+y)!}{x!y!{\left(1+\frac{N_{2}}{N_{1}}\right)}^{\left(x+y+1\right)}}$$

(N_1_: the total clean tag number of sample 1; N_2_: total clean tag number of sample 2; x: tag number of gene A in sample 1; y: tag number of gene A in sample 2). The criteria used for assigning significance were: *P*-value < 0.05 and estimated absolute |log_2_Ratio(treatment/control)| ≥ 1.0. Three replicates were used for analysis. To clearly visualize the expression profile data, a heatmap was generated using Cluster v3.0 and Treeview software.

### Validation of the expression of miRNAs by stem-loop qRT-PCR

A well-developed stem-loop quantitative real-time PCR (qRT-PCR), which could distinguish single nucleotide deference between miRNAs, was performed to validate the expression of miRNAs identified by sequencing. It includes two steps: a reverse transcription reaction and a real time quantitative PCR reaction. Reverse transcription reactions were carried according to Chen *et al* [44]. The samples were collected as described above. All primers involved are listed in S6 Table.

PCR was performed using a Eppendorf AG 22331 Hamburg thermocycler in a 20 ul volume containing 10 ul SYBR Green PCR master mix (TaKaRa, Japan), 0.2 uM of each primer (S6 Table) and 10 ng cDNA, and the amplification programme including an initial denaturation at 95°C for 60 s, followed by 40 cycles of 95°C for 15 s, 60°C for 15 s and 72°C for 20 s). At the end of the cycling process, a melting-curve analysis from 55 to 95°C with a heating rate of 0.5°C s^-1^ was performed to determine specificity of amplified products. The chrysanthemum *EF1α* gene was used as a reference. Relative expression levels were calculated using the 2^-ΔΔCT^ method [45]. Three independent biological replicates of each sample and three technical replicates of each biological replicate were used for qRT-PCR analysis.

### RLM-5’ RACE

RNA ligase-mediated 5’ rapid amplification of cDNA ends (RLM-5’ RACE) was carried to map cleavage sites in the predicted target genes of miRNAs according to Llave *et al*. [46] using a GeneRacer kit (Invitrogen, Carlsbad, CA). mRNA was purified from total RNA extracted from leaf tissues using PolyATtract mRNA Isolation System IV kit (Promega, Madison, WI, USA) according to the manufacturer’s instructions. The final PCR products were gel purified and connected to pMD19-T (TaKaRa, Japan) for sequencing. At least 15 independent clones were sequenced.

### Accession numbers

The data sets supporting the results of this article are available in the NCBI Sequence Read Archive (SRA) database under accession number SRP051318, http://www.ncbi.nlm.nih.gov/sra/?term=SRP051318.

## Supporting Information

S1 Fig The expression of *ARF* gene (Unigene29593_All) in response to aphid feeding. CK: control; A: aphid infestation treatment.
(TIF)

S2 Fig The expression of *TIR1* gene (CL1261.Contig1_All) in response to aphid feeding. CK: control; A: aphid infestation treatment.
(TIF)

S1 File The precursor sequences of potential novel microRNAs (miRNAs). (DOC)

S1 Table Summary of conserved microRNAs (miRNAs) identified in library CK, A and M. CK: control; A: aphid infestation treatment; M: mock puncture treatment. “-”: no hit.
(XLS)

S2 Table Differentially expressed conserved microRNAs (miRNAs) in CK-VS-A, CK-VS-M and M-VS-A comparisons. CK: control; A: aphid infestation treatment; M: mock puncture treatment. CK-VS-A: comparison between library CK and A; CK-VS-M: comparison between library CK and M; M-VS-A: comparison between library M and A. The expression abundance of each miRNA was estimated by std, normalized expression level of miRNA in a sample. If the original miRNA expression in a library was zero, the normalized expression was adjusted to 0.01. The criteria used for assigning significance were: *P*-value < 0.05 and estimated absolute |log_2_Ratio(treatment/control)| ≥ 1.0. “-”: no statistical significance.
(XLS)

S3 Table Targets of the differentially expressed conserved microRNAs (miRNAs) in CK-VS-A, CK-VS-M and M-VS-A comparisons. CK: control; A: aphid infestation treatment; M: mock puncture treatment. CK-VS-A: comparison between library CK and A; CK-VS-M: comparison between library CK and M; M-VS-A: comparison between library M and A. GeneIDs were got from the Chrysanthemum Reference Sequence Database.
(XLS)

S4 Table Summary of potential novel microRNAs (miRNAs) identified in library CK, A and M. CK: control; A: aphid infestation treatment; M: mock puncture treatment. “-”: no hit.
(XLS)

S5 Table Differentially expressed potential novel microRNAs (miRNAs) in CK-VS-A, CK-VS-M and M-VS-A comparisons. CK: control; A: aphid infestation treatment; M: mock puncture treatment. CK-VS-A: comparison between library CK and A; CK-VS-M: comparison between library CK and M; M-VS-A: comparison between library M and A. The expression abundance of each miRNA was estimated by std, normalized expression level of miRNA in a sample. If the original miRNA expression in a library was zero, the normalized expression was adjusted to 0.01. The criteria used for assigning significance were: *P*-value < 0.05 and estimated absolute |log2Ratio(treatment/control)| ≥ 1.0. “-”: no statistical significance.
(XLS)

S6 Table Stem-loop qRT-PCR primer. (DOC)
