# Supplementary figures and images for: MicroRNA Expression Profile during Aphid Feeding in Chrysanthemum (Chrysanthemum morifolium)
Source: PLoS One. 2015 Dec 9;10(12):e0143720. doi: 10.1371/journal.pone.0143720 (PMC4674109; doi:10.1371/journal.pone.0143720)

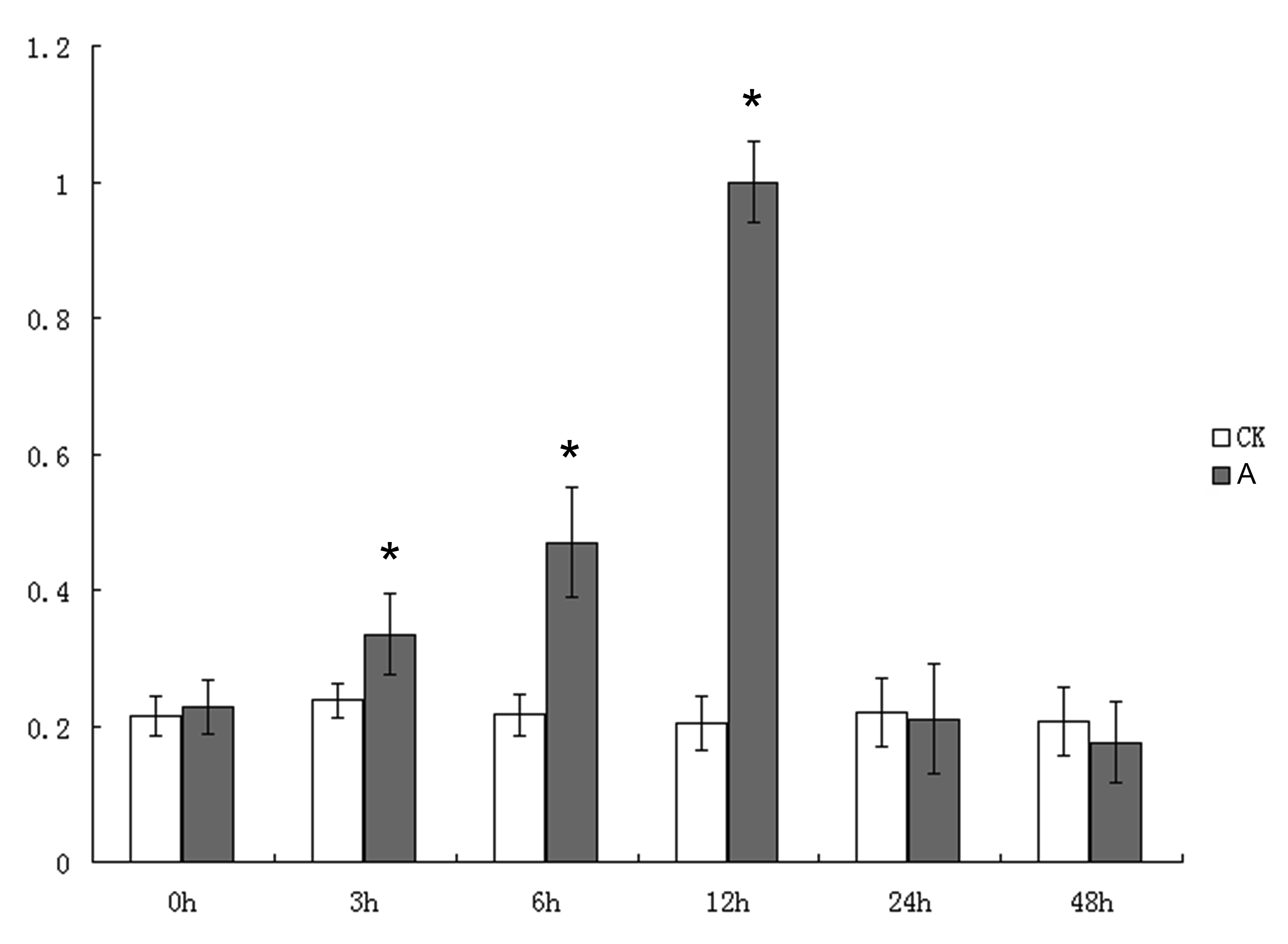

Supplement: S1 Fig — CK: control; A: aphid infestation treatment. (TIF) [file pone.0143720.s001.tif]

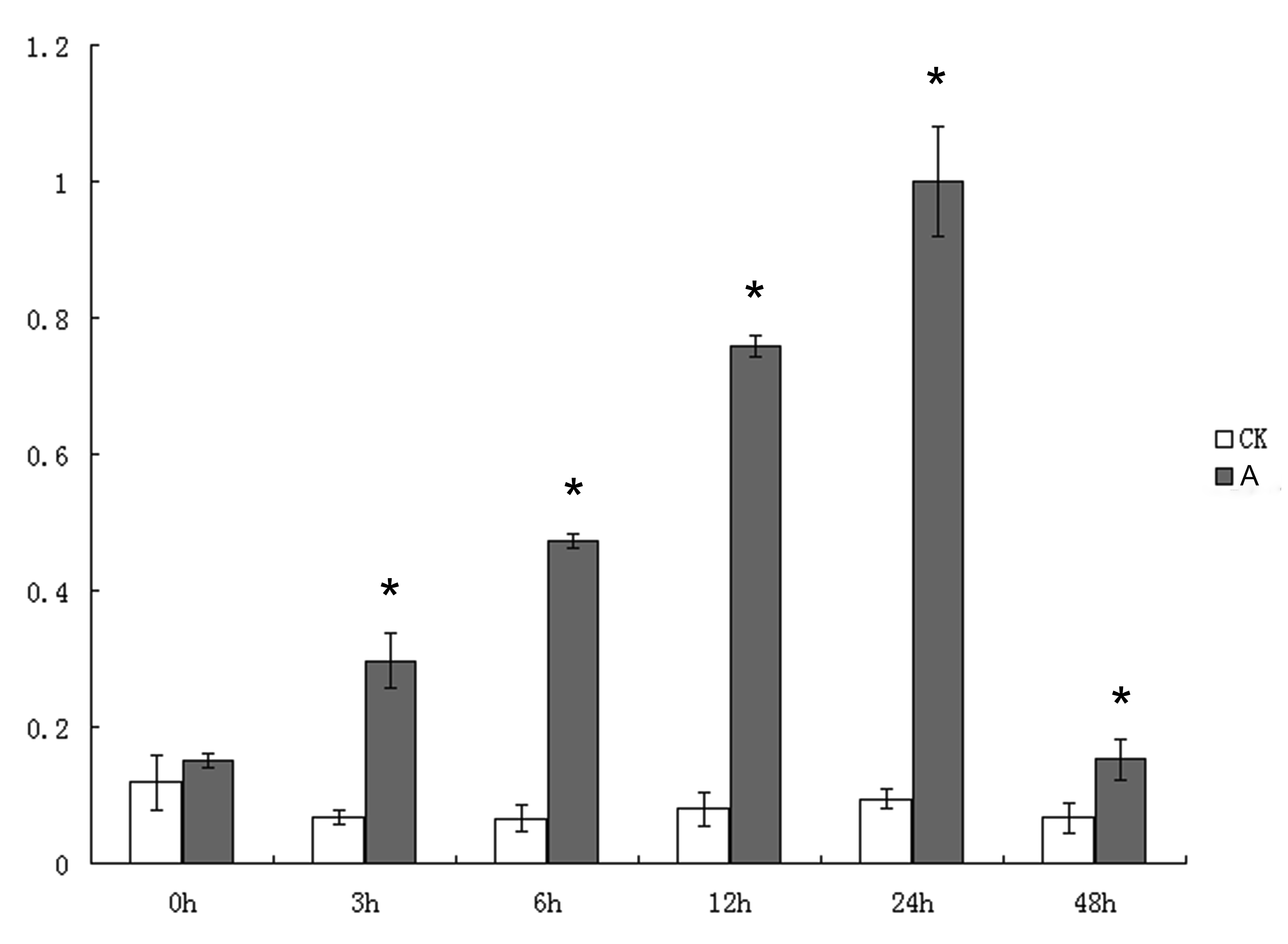

Supplement: S2 Fig — CK: control; A: aphid infestation treatment. (TIF) [file pone.0143720.s002.tif]
