# Supplementary material for: MicroRNA Expression Profile during Aphid Feeding in Chrysanthemum (Chrysanthemum morifolium)
Source: PLoS One. 2015 Dec 9;10(12):e0143720. doi: 10.1371/journal.pone.0143720 (PMC4674109; doi:10.1371/journal.pone.0143720)
Supplement: S1 File — (DOC) [file pone.0143720.s003.doc]

S1 File. The precursor sequences of novel microRNAs (miRNAs). Red: Novel miRNA sequence.

novel_mir_1:

GATACTACCTTAATTCCGAGACCACCTATTTCAATGATGATACACGGTTTCCGCTTGTGTGTCGTACAATAACAGCAAAAATAATAAATATGGTTTTGAGCATGTGTGGTGTG

novel_mir_2:

CCCTAACATTATTGTTATATATTACTCTTTACCTAAATGTACATTTCACAAGTCCAAACCCTACCTTCATATAGATCTGCCTCTCTGGCTGACTCTTCACTAGTTTTTTAATGCAAGCAAACAGAACCAGAATCTTCAAGGATCCAAGGTCCAGTCGGTCACATTTGCTTCAAATCCATGATTTCATTTGGTTTTGTTCTATGGACGACGAGGATCTTTGTCATGGATATAAAATATTGGCCACATGTTCTCGAGTTTTAGAGGTGACTTATAGCATTGATGTCATTG

novel_mir_3:

GGGGCTTAAGGCTTATTTGGTTTTCCATAAGCTCATTTTGTGCGGCTTATTTCGTAATCTTTTGGCTTAAATAACCTTATCGTGTCCTAATAAGCTTATATAAGCCGAAAAGTTTCAAAATAAGCCACAAAAAGTGAGCTTATGAAACATAAGCCCA

novel_mir_4:

GCATCGTTAGTCCTAAGGACTAACGGTGCAAATGTTTACTCTTCCGTTAGGAGATAACGGAGAAATACTTGCACCGTTAGTCCTCTAAAACTGAGGGATGTA

novel_mir_5:

ACAGGGAGTGGCACCGTTTAGTATCCTAAAATTAGTATATGTATAATGAGGCTCAGATTTAAAGAGGATCGAAAAAAGAGGATACGCCCACCATGGCTGCAGTATAGTAACTTGCCGCGCCAAGATATGGACCATCTCAGCTTTAATCCCCTTGCCACCATAAACTCATCATAGCCTGTTGTAATATATTAATTTTAGGATACAAAACGGTCCGTACGTG

novel_mir_6:

GGTCCCGACACTGTTCATACACAATGCTTAGTGTTTCATGCGCCGAATTCTGAAAGCTATATCAATGCATGCATTGATTACAAGTTGAACCGATTTCCAACTGGTTGTTGAATCAATCAATTGATTGACTGGATAACCACTTAGAAATCGATTCAACTTGTAATCTTTGCATGCATTGATATAGCTTTGAGGATTTGGTGTATGATACACTAAGCATTGTGTACGACGACCCCCGGGACA

novel_mir_7:

AAGCATCAACTCTTGGATCATTAGAAGCATTTATCGTGTTTTTAAATTCACAAGATGCAAATATACCCGTTGGTGATA

novel_mir_8:

GGTACAGTTATAGCTGCTGCAAATACAATTGTGACAATTAAAGCAGCAGTGATGCTACATGACTCGGCAGTGGTTTTCATCCACTGTTCCCCTTTCTTCAGCAAATCCGCATGTTCCCTTGTAAAGACCATTTCTGACGTATCCATATCGATATTCTTATACGTAAGTTCTATAGGGAGCATGAGCTTCTTCACTTCCTCATGCCATTGGAGTTCTCGTTGTAATTGCAGTGCAGCACCTGTAGTACGACTGAGTACAATTGAAGATGCCA

novel_mir_9:

TAATCGACAAAGGGGTCCTGGAGGGGTGTGCAATAGGTGCCGTCACACAAAGCCTCCACTTAAAAGGGGCCACATTTTTATTAATTTTGCATATAAATACATATAATATTAAACATACACGAGTCATACAATTTAAGTTTATATGCAATTTCTTTTTAGTTTACAAGTATAATAGGTTTGTTTTGTTGTTATCGCCTAGGGCCACCAAAAACTATGA

novel_mir_10:

TTACAAAGGATTATGAAAGTAGTCTAGACCACTTGCAACATCAATGCATATCTTGAGTCATTAGTTCACATGAGACTTGTGCTTCCAAAAGCTTGTGTTGCACTTGTCTCACGTGAGAATCTAATGACTCTAGATAGTCATTGGTATTGCAAGTGGACTAGCTTACCGTCATAATCCTTTGTAGT

novel_mir_11:

ATACGTGATCATGCGTTCAGGGGGTGACTTAGGTCATGACGGGTCACAGGATAATCTTTAAGAAGATCATCGAGATATTAATAATAATAGCATCTACTTACAATATCATCTATCCAATAATAACATCTACTTACAAGGAAATAATATAATACCTGAGAAGATTTTTACCAATTACAATATCCGGATGATCTTCGTAATTATCCAAGGGTGTTACCCCGTCATGACCTAAGTCACCACCTGCACGCATGATCTGTAG

novel_mir_12:

GAACGAGACGAGATCGTCGAAATCGACTCGAATTTATTCGAAAGTAGTCGAGCTCGAGCTTGGCAGTACTCGGGCTCGGTT

novel_mir_13:

GGTGGTTTTTCTGATTCGCTTGGTACAGGTCGGGAACTGCTTCGCCGAAAAACATGTTTTCTAATAAAAAAAAGTCGGTGAATATTAAGTCCCGCCTTGTATCAACTGAATTAGAAACCAC

novel_mir_14:

ACAATTACATTGTGGATATTGAGAAACTGAAATGAACTTGTACCATCTCAAGATGAAGAATAGTGCAAGTTCGTTTTAGTTTCTCCATGTCCACAACGTAATTG

novel_mir_15:

ACTTTGGCTGTGACCGACAGGAGCTACCTCGTAATTCAAGCACGCTTTACAGATGTTGTAGTCTCGTCATTTATAATAATAGACCTAGACCACAACATCCGTAAATGTGCTTGAGTTACGAGGTAACTCTCGTTGGGCACGACCAACAT

novel_mir_16:

GGCTAACGGTAGCGACTGCTGAGGAGATCGAGGCCATTTTAGTGCTTTAGATGATTAGCCTTAGCGAATCCAGTGAGTAACTTTAGTTTACATAGCTCTATATATAATGGTCGTATATGGGCAATGGGTTTGCTACTTGCAATCCAATGGACTAAAATAAATCAAGCTTGACTTCTAGTGATTGTGGCCGTTGGAT

novel_mir_17:

TAAAGATTACTATTTAATCGAGTGGTCTGTCATGTATTGGTGTGCTAAAATATGTGAGTTAGAAACTCAAATACTTTAGGACACCGATACAAGGCCGACCACTCGAATAAATAGCTATCTTTGAA

novel_mir_18:

GCTTACTTTTTGTGGCTTATTTTGAAATTTTTTCGGCTTATATAAGTCTCTTTGTGTTACAATAACTTTATATAAGCTGGAAAGTTTCAAAATAAGCCACTAAAAGTGAGCT

novel_mir_19:

CCCCTTTGAATGGGCTTGGGCTTATGGAGGCTTATGGCTTATATATAAAAGCCTCCATAAGCCCGAGTGGCAAACAAGGCTT

novel_mir_20:

CTTTAAAGAACTCAGGGTCTCCTTGCCCATATTTACGATCTAGCCACTTTAAAGCAACCAAGCTTCAGCCCACCGCTGGTTCTGGTAACTTTGCTGTTAAGTGTGAGCGGGTCAGGTCAATCTGGTTAAGAGTTCTGAGTTGGTGAAA

novel_mir_21:

CCACTTTCACGTGGACATAACTTCTCAAGATATGCCTTGATGCTGCGAAGGGACTAAGCTTGCATTAAAGCATATCTTGATACGTTGCGTCCACGTGAGAGTG

novel_mir_22:

CAGGTGGAAGCTTCCAAAGGGATCGCATTGATCCGTCTTATTAATTTTAGTTGATGTTTTGGATCATGCGATGTCTTTGGACGTTTCCATCCA

novel_mir_23:

CAAGGATCTATTTGGTACGAGTAGTTTAGATGAGGGGGTGCCTGTGTCTAGCATATCTTGTTTAAATGGTCTTGA

novel_mir_24:

ACCCGGGTCGGGTTCTGGTTCTGGTTCGGGCAAAGGGTCGTCTGATAAGCTGTCGGGTGGGGCGATTGCTGGTATTGTGATAGGATCTGTATTGGGTCTTTTGTTAATTTTGTTAGTTTTATTCTTTTTGTGTTGTAAGAAGAGAAAACAAAAGGATGTAGTGGCTACAAGAGATTTAGGAGAATCTAAACAAGTTGAAGTTGAAATACCCGCGTCTGGGCCGAAATCAAAATCCGAGCCCGGGG

novel_mir_25:

GTAATGGACAAATGGTGCTATTGTATATTGTCACTGTATTTGGAGAAATACGGTGCCAAAATACATTAGCAACATTTGTTGATTACA

novel_mir_26:

GCTTATTTGCTTTTCGGCTTATATTTCATAAGCCCTAATTTTCCAGCTTAAACTTGTGGGCTTATATAAGTTTAAAATAAGCCGGAAAATTGAGGCTTATGAAATATAAGCCGAACCAAATAAGCC

novel_mir_27:

CACCATGACGCACATTACCAAAAAGGGTCAGCAGTGCCTTATCATGTGGTATCCAGTCAGAACTATCCATCATTTTTAAGTCTTCTCTTTTAGGGTCTATAAGTCTTCTCCTTTTTGGATCCGGAAAGTTTGATGAAGATTGTGATTTTTCTCCTTCTTGAGATCTTTTTCTGGTGTTGTGGGTTCCCATT

novel_mir_28:

TTTATATCTATGTCCACTGATGAAATCGGCGTCGGGCATGGCCAAAGTCAATTTTATCAGCGGTAATATAGATATAAAA

novel_mir_29:

TTGGCTTTTTTGAATTTGGAGAAGCTCATAAGCTCATTTTTTTCGGCTTACGAGCTTATCGAGTTGAGGTTATGGTTTAAAAAGCCAGAAGCTCAAGTTGATAAGCTTAGAATCGGAAAATATTAAGCTTAGAAGTTCAGTGTATGAGCTTTTCCAATTTCAAATAAGCCCG

novel_mir_30:

GCAGCGGGATTGCTTTTGTAGACGGCAATTTGGAACTAAAGATCGGGGAAGTGAGTTGTATATACCATTGTCGCTCTATAGTATGAAGAAGACCAAACCATTGTTGCATCTGTAACATTTCTTCACTGGTGGGTTGATGAGTATTCCAGAAGTTCCTTCCATATCCGATGAAATACAGGTAAATATTGTCCCTTTCAACCACGTATCGAGTATCAAAGGGGTTGTGCCAAAGTTTCTTGCATGTG

novel_mir_31:

TGAATAGATATGGAATCCAAGTAGAGTGCCACGTTTCTATCAAGAAGAGAGAGTGGGTTCAAGACCTTCCCTTTGTACATCTATAAGAAGGTTGGTTTTTCAGTAGAAGACTCCATCCATTCCTCAAAATAAATTCAGAAATTAGGGTTCTTAATAATTAGGGTTCTATAAATCAAAGGGCTTCCTGAAATTGATGTGGGGGTTTTGTAACTGGAAGTATAAGGAAGCTATTCCAAGTATCAAATCGTGCCAACTCACCCCGAGACTTCCCCAATTGGAGAAGTGATGCCGGTGCTCAATGCTTTCTACCCACCAATTTGTTTT

novel_mir_32:

ACAATGACCTCCATCCATTTTTCTCCTTCAATCACCAAATCCTTGTGTTCTCTAGTGCATGCCATTTTAAAATAAAATGATGTTTACAAGAAAATCCAAGGATTTGGTGATTGAGGGACAATAATGGATGAAGGCTATTGC

novel_mir_33:

TAGGATATGTCGAGTTGACAGAAGAGAGTGAGCACACAAAGTTAAATTGTACATAAATGGAATTGTATGATTATTTTTTGTGCTCACTGCTCTATCTGTCACCTTGTATTCT

novel_mir_34:

TCTCTCATAATGGGGGACGAGCATCTCAGCACTATTTCGGAAACGAAATTGTCTAGTGTTGAGGACCTTGTCCCAATCCCAAGTGAGTCTGAG

novel_mir_35:

CTTTGCAATACCTGTGAGCAAGGGATTGTGGCGACCTCAATGCACAAAACATGTCTAATTGGAACTCACAAGCCTTGCAAGCATAGCTTATACAAGCAGAC

novel_mir_36:

AGAGTCAAGGTTTCCAAAAGTAGCAGAGATGTTTATTTTTTCGGATTTGAAGATCAAAATCAATATCAGAACTCACTTTTTATCTCTGGGAGTCAACTACGGGGTTCATCTGGTCTTCAAATTCTGTGGTCCAAGAAAATCTTTGAGTAAATCTTCGTATGTGAACCTAACGTATAAAATGGGGAAAGAAACCCTCCATGCATATTTCGCTACATGGAGAGACGAA

novel_mir_37:

GATAATTAAACACTCAAATATCTAGTTTGTGTTTAAGTAATTTCCATTAATATTCTTCTCACACATTTGACCACCTCAGAATTGATATTCTTCTCACACACAAGCTTGAGATGTTTGAACAACATAGCCAG

novel_mir_38:

AATCTAATACTAATCAAATTGACTAATTTGACTAACCAACTCGAAGAAATAACTGTTCCCGTGTATTTGTAAATACTCAATTCCTGGAGACCCGAATTTGGTTCTAATCCCTCAAGAACCTCTTCATCAAATGTTTCTGATGTATCATTCCTCGACCACAATAATGTCAAAGCCCGCATATTTGTTTTGTCTTTCAAATTGGCACTTTTGGCATCCCTTAAACC

novel_mir_39:

TAAGCTCATTTTTTGTGGCTTATTTTGAAACTTGTGAGATTATATAAACTTATCATGACATGATAAGCGTATACACAACAACAACAACATGATAAGCGTATATAAGCCAGAAAATCTCAAAATAAGCCACAAAAAGTGAGTTTAT

novel_mir_40:

TGGCGAATTTAACGGAGTAGTGTAGCATGACGTATTAGTACTATTATTGTTTGTATTATTTCCATGTATACATGATTCTGGCGAAATTTCGCC

novel_mir_41:

CCTTTCTTTTTTTGAAGGGAGTATTGCATGTTCGGCACTTGTGCTTCAACTTTTTTGAACTCTTTTGTGCATAACTTGTAACACTCCCTTAAAAAAAGAAGGT

novel_mir_42:

ATTCTTTTAGTCTGAGCAGCTTTTAGTCTGCACAGGGCATCTTTATTGGCTTCAAACATTTGTACAAGGCGAATCGGAAAGAACTCCCGTTTGCTGCTGGGCAAGTTGCTAAGGTGTTCAGGAATGAGGTAACATGCTCTTTTGAATAACACCTAACTTTAATGTCATTCATAATTACCTTACAAGTGTAGAGATTGACTCTCTTTAACCTATTTTTTTTGACATTTTGAGATATCTCCTAAACAAAAGCTGCTCAGACTAAAAGAATTTAAACTTG

novel_mir_43:

TGGTTGACTATTGATACGGCTGCGAATCAAGGACACGTTGTTAGGGTCATCTAATAGACAGCCAACTAAGTCCTCGACAAATAGCCTTGTCTCGGATAGCCTGTTCTGTCGAGCT

novel_mir_44:

TTAAAAGCAATTGATGCGGCTGAGTAGGTCGGGTGCCGTAACTAGTGATAAACTCAGAGCTGTCGATTGAAGACTT

novel_mir_45:

GTAGCTTTCTTGACCTAGCGTGTAGGCGAGTATTCATTCCTGGCTCTAGTCCTCTTTCACAGTCGCGCTTCAAAGCATGCTTGTGAAGGTCAATGGTGATAGAAGAGCACTTGAGGAAAGTATAGGTTGTCCCTTTTGTGTTTGACCTTCAAGAGAGACAAATAATTTCCCACTTTCATGTTTATGTGTTCTGCTTTGGGGGAGACGAGGAAACAGAAAAGAGAAAAAGAATGAGTAGAGTGGCGTTTGGTGGCGTGACTGTGGAAGAAAGACTAGAGCCAAGAAAGTGTA

novel_mir_46:

TATCGCCTTGCTGAGATTTGGGAAAAGACGATAAGCTCACTTTTTTTATGTTTTCACCTTCTACTCATGGACTTAAATAAGCCATGAGTTAGAGATGATAAGTTAATAAGGCGAGGAAAGTGTGCTAATCGGCTTTTTCCAAATCTCAATAAGCTGATAA

novel_mir_47:

ATGAGAAAGTTATTGTTTATGAGTATGCATCGAGAGGAAGTCTTGATAAGTACTTGGACAATGATAGTCTTAC

novel_mir_48:

CCAAACGTCATTTGTTTTGTGGATGCTGAAGGCAGTACAAATCACATCGTCGCTTAGCGAACCATAGATCAGTATGACAAGTGC

novel_mir_49:

TAGTGGGAGTCGCATCGTGGAGATGGACTACATTTTCGGTACATACAAATCGAATCCATCATGAATACCCCAGATGGTTGTACCGAAAAAGTAGTTCATCTCCACGATGCATCATCCAAGTTG

novel_mir_50:

AAACAACAATGCCACGTCATCATTCCGTACAATCAAAGTTTTCATTTTACAATTCACCTAACAAAAAACCATTACCATGCCGTACACTCTCCAAATTAAACTTCAAAATGATGACGTCATCACTCAACCTAACCTCGAATGTGCCTGCGTGGCCGGAATATGCGGGGCTGCATCAGCAACATAGCGTTGAGTATTGGATGATGACGTCATTGCTGTGT

novel_mir_51:

ACCATACACGAGCTGTCACAACAGGTTCCATATTCATGGTATACTCCTTTAGCTCAAGGGTTGTACCTTGCATATGGACCTTGTTGTGGCACCTCGTGTTTGG

novel_mir_52:

GAATGGTTTTTTGTAGATGCACATGTAGAAGCTTTTTTGAGTTTTACGTGTTGTTTCATCGAGGTTCCGATT

novel_mir_53:

AAAAGGTTGGTCCAAAGTCGTTAGTAAGCAGCGCTTCCGGACAATAACAGGTGACCAAAACTGAACAAGCACATGTGAGTCTCGGAAACATAAAGCCAAAAGTGCAGTCGTGATTTTTTCCTTTATTATTTGTGGGTTTATGATATTTTGATCAGCCAGCA

novel_mir_54:

GATTCTTATGCTTGAGAGTAGAAAGCATGGAAATCTCCATCCAAAACTGTTGTTCTCGATCCTCCATGTCTTTATTTAACCTCTGAGCACGGATATCA

novel_mir_55:

ATCAGTAGGTTCATGCCTGTCAGAAACAATATTATAGATTTTAAAGACAACTTTACGGTTTGGCCATCGTTCAGATAGTTCTGCTGTGTAAAGTTTACTACAAACATGAGGTGGGAGAGAACTCTCTTTAGACATTTGCATGGCCAATTCGATCTCCTTGAATGGAATACGTATTTCTCTTGGGTTTCGATATTCACTGCTGCTTCCTTTGACAAAATTAGCTTCCCGGGTATGAATTTCCAATGCATCTTTAATTGTCATGGCGAGCGTATACATTGTAGGGCGATCCTTGATGTTGTAACTAAGGCATTTACATGTGA

novel_mir_56:

CAGACTCACGATGCATAGCGTCTGTTGCCAAAGCATCAAGAATATCATCTATGTCGTAAGCCAAATGTTGGAGACCATTCAGCCACTGTTTAACAGCTTCATCTGTTACTTCTTTCTGAGAAGCATCATTAAGCAAGGCTCGGATCTGGGGTAACTTCATCTCCCATTTCTTCAGCTCGGAATGAATTTCTTTGGAGCGGGCAATCTTGTTCATGGCTGCAGATGTTAGCTTCTCAAAAATG

novel_mir_57:

GGTCGGTCGTAGGGGTGTTATCGGGTCGGGTCGGGCCGGGTTTTGGTTAGAAAAAACTAACCCGCAACTCGACCCGATAAGGAGTCGGGTTATCGGGT

novel_mir_58:

GCCTTTCCCAATTTTGAACCAAGCGCACCCTTAATATAACCCAACCCTTTGGGGTTACCTTTGCTTCAGGAAGGGAAATCAACTGGCA

novel_mir_59:

TGGTCGGGTTGACCCGATAGACCCGATAAGCCGAGTTGAACCCGACCCTATAAGGCTTTCTCGGGTTCGGGTCGGGTCGGGTTGGCCCATTTG

novel_mir_60:

GACCTCGTTCGTGTTTTTGATCTCTGCTTCAGGTGGCAAGCCTACTTGGCTCCAAGTCCTAAACATCCGGCTGTACTCTTTGGCACAGTATTTGTTAGTTTTATGTTCGTTTAAAGAACAGTTGCAACAACATGTCAGAGTTCATGCTGTTGAAGATTTCATGGCATGCCGTGAAATCAAACACAACTTACAAGCTCTCACTGGCTTGCGACCTGAAGCGGCAAGATCGAAAACGCGAACGAGGCC

novel_mir_61:

TATTAACACAAACACTATGTTTCGTCCCGTAATCGCTATCTCCTCATCAATCTTCCTCCACTCATGTTTTGGCATCGTAAAACATAGGGATTCAAAAAGGTTTTAGCGTTTGATTTGAGGAGGGAGATAATTAAGATACGAGAATGAGGCGTTGCCAATTGAGTATAGTTCGTGGTATGATTGTAAAGGTATGCGGACGTTGAAGTTTAATCAGCCGTTTTTTATGAAGGTGAATGGGTTTTTGGAGGTGCTTTGTCATGACCTGGTTGGCTGGTTGTGGAAAGTAATGAGATGGATATTTGGATGTTGAAGGATTACGAGGAGCGTGTTTGGGTTAAGGAAACGATTGTGTTTGGTGAGTC

novel_mir_62:

ATATGATACATGACAGAGGAAGTATGATTGTACAAGAAGATAGAATAAGTAATCTGCCGCATGGATATTATCCATCCCATTTTCCCGGTCCTTGAGATGATATATGTTATTCGAACTAGTGCAGTGTCTCGGAAATGAAACCTTATTTGGACTTGCAAGTTGTTTCACATTGCTAAATTTGTAAACCACGCACATTCTCACCGCAACATCCTTGAAGACACTGTACAAGTATGATTAACATATCTCATGTCAAAAAAGGAGAGAATGAGATGGATAAGATCCATACGGCAAAGCTTATTCTATCTTCATATGCAATCATACTTTCTCTTTCATGCATCATAC

novel_mir_63:

CTTTGCCGTATGGATCTTATCCATCTCATTCTCTCCTTTTTTGACATGAGATATGTTAATCATACTTGTACAGTGTCTTCAAGGATGTTGCGGTGAGAATGTGCGTGGTTTACAAATTTAGCAATGTGAAACAACTTGCAAGTCCAAATAAGGTTTCATTTCCGAGACACTGCACTAGTTCGAATAACATATATCATCTCAAGGACCGGGAAAATGGGATGGATAATATCCATGCGGCAGAT

novel_mir_64:

ACTCACTGTGGCCTCTGACTTGCTTGGTACAGCTCGTGAACTAATTATCTGGCTGTGATGTCTCGTGTACACGGATTTCATGAGGATGATTACTAGTTCCGGCTGTACCAAGCATGTCCGAGACGCAGTGAAAGAA

novel_mir_65:

TATACGTCTTCAAAACACCTTAATGGTGATATGAGAAAGAAATGAGTGTTCATTTGTAAACCCTAGTCACTACTTCACTCACTCACTCACTATCTTTCTCATTTCTGAGCCGCCACCTCCTCCTCCTCCATGGTGCCGCCGCTACCACCGTTTGCCACCACCACTCACATCATCATTAAGGTGTTTTGAAGACGTATAACAAAATGTCGGCG

novel_mir_66:

CCAAACGTCATTTGTTTTGTGGATGCTGAAGGCAGTACAAATCACATCGTCGCTTAGCGAACCATAGATCGGTATGACAAGTGC

novel_mir_67:

TTATGATGTCGATAAAATACTTCCATGGTTTTGAAACGGACCTTAAATTCGATTTAGGTCTGTTTCCAAACCATGGAAGTCTTTTGTCGACAGCTCTTGG

novel_mir_68:

TAAAAAGGAATTGAGATCCTTATGTTCAAAAGGTCATGTTGATCAATTAGAAGCAAGCTTGTTAGTAAATCAATTGCCCACTTTTTTAGTTGCTATCATTTACCAACATGGCTTCTCATGTTCTCACTTTGAACATGCAAGGATCTCTTTCCCATCAA

novel_mir_69:

GTGGGTGGTGCCATGGAAAAAAGTGGTGCCACATCAGCACTTTTTCTCCATGGCACCAGTTTTGGCTATGGCACCAAAACCCA

novel_mir_70:

CCTCTTAACCGGTCAGACGTTTTGGATGTTCTGACCGTTTATTTTTTTTCCCGTTCAGACGCGGTCAGAGGGAGTTACATCTTAATGGTCTAGACTTGAGAG

novel_mir_71:

ACACACATTATGCAGAATGGCAGAAGTCTGTTTCCTACAATACAAAGTGATCCCTAACGTTTGAGCGACATTGGGGTGCTGCGAGTGATCAATTCCCATGTCTTCCTTAGAACTTTGTGTTTAAATGGGCACTGCCATGGCCATAATCACCACCTAGAGGAAGAACATAAGTGTAC novel_mir_72:

TCATTGTGGGTGGAACTCGACTTTGGAGGCGTTGTCAAGCGGTGTTCCCGTGGTGGCATTTCCGGGGTGGGGTGATCAAGTGAC

novel_mir_73:

AGGGGGAACATCATCCCTTGCTAGCAGCCATGTTGCCACAAGGTAATGAGGCTGCTGATGAGGAGAATGTGTCTAAT

novel_mir_74:

ATTCTTATGCGGAGACGATCTGGATTGCGGCTTCTCCCCTTTGCTTCTAACCCACGAACCAAATGGCTACGTCAACCTGACGACCATTTTTTTTGTAGTCATTAACGCCTGCTGAATGTTGCTGCTGATGTTCTTAATAATGATCAGAACGCGATTGGCCGTCAGCCTATAACTGACAATTGTTTGCTTCCAAATATCTCTGTCATTCGGCTTTGTTTCTCACAATCATAGGCCGAGATCAGACGCCGAGTCAGGATGCCTTTTGCATAAAGAATC

novel_mir_75:

GAGGGAGTTTTTGACTCAGATGTACGCGGCCTAAATGGGGTATCCCAGTGATCGTTTCCGGCCCAGTTGTATTTATATATTTAACAAAATACAATATAAAATATATATTTATGTGTATATTTTGCTTATCTGCAGACATGGAAAATACATCACCCATTGCACCGTTCGACCAACCAGCTTTGTGTGCTTTATGTCATAGAACACTAACTTCTGATAATGACCCTATAGACCTCG

novel_mir_76:

TCAAATTGGAGATGAGGTGAGATGAGTGGACCCTTTCCATCTTCTGCAACGATCTGATCTGATTATCGAGATTGGATAAAGGATGGTCGAAAACCTGGAAATGGCAACGAACGACGGTGGCTAGCGGTGGTGTTGACTTATTTGACTTGTATCTGATAAATAT

novel_mir_77:

ACACAGTATCCCACAAGTAGTGGTTCTGGTTCGGGTTCGGGCATTGCTTTGGGAGAGCCGGTGG

novel_mir_78:

TTTTACCGACCTCGGAGAAAGAGAGTGGCTCACAGTATGTGATGACGTTGGTTTCGTTACCGCTGTCTGTGATCCACTCTGCTTTCTCTGAGGTCAATACGT

novel_mir_79:

TCAAAGATCCAGGGAGATCATGGTAGCTTAGTTTAAGAGCTGGAATAATTCCACCTCCCTCTTCTAAGCTCCATATCTGACTGTCCTCTATTT

novel_mir_80:

ATCTTGCTGGTGCTGGCTCGGGATTGGTCGGTCAACTCAGTATGCTTAGCCTCGGTTGCCAAGTCATCAAGTACATCATCTATGTCATAAGCCAGATGTTGGAGATCATTGAGCCATACTTGAACAGCTTCATTAGTTATTTCTTTATTCCAAGCATCATTCAGCAGTGCTTGGATCCTGAACAAAGTCTTCCCTAGTTTCTTCAGCTCAGAATGAATTCCCTTTGCTCGAACAACTTGCTTAACAGCTTCACTGGCCAGCTTCTGAATAACAGCTTCAATGAGGG

novel_mir_81:

AGGAGGGGACAAGGAAAAGGTGAAGGTGAGCTAGGGGATGGGTGGAAGATGTGGGAGATATGGAGCAGTTGGGAAGAAGACTAATTGGCGCCAAGTGGGAAACCAGATATTTGACTGCTTGGGTGTTGACTGGATATTAATACAAAGGGGTTCAAAATACATATATGGCAGGGGGTCAAAGGGTGAGAGGGTCCAAAATAGATATTAAGAGTTTATTAATACAAGGACTTAATATGTAATTCTGAAAATTGCAAGGACCATAGCTGCCAATTCAACAAAGTTACTTCCAAGGACTAAAAGTGACATTCTCAAAAAGTATAAGGACCAAAATTGCCACTTTGCAATTTTCCAGGGACTAATTCCG

novel_mir_82:

GGTATTGTTTTCAATGGATCTGTACGATGGAATCCTACTTGTAGTTTCATCGATTCAGATTCAGGCATGATATTTTTCGACCA

novel_mir_83:

GAAATAGATCAAGGAGTGATAGTGCACGGCTTCCGACACGCAACATAAATTTTTGGTGGGTCGAGTGGATTAAAACCGAACACACAAGAGCTCCAATCGGCACGAGTCGGAAAGAAATTGGACAAAATGGAACTTGATTCATGCCTTCTAGACCATGAATAACAAAACCCCTCTAACAATTTTCAAAAGCCCCTTGATTCGTGAAACCCTAGTTTCTCGAATTTTATTTATGTGGAAAGATGAATGAAGCTGTGATTGCTTACTCCCATATGTAGAAG

novel_mir_84:

AATTTGCAGGTTTGGTCCCTATTCTTTCAAAATTGCAGCGATAGTCCTCATTTTGAAGTTTAGATTACATGTTTGGTCCTTGGCTAAAGTTAAATGACAAAAATACCTTTTATTTATCAAGAAGACATGGGTAATTTGGTCATTTCACATTGATAGGGGGATTGTATTTACAATCCAAACTTCATAATGGGGCTACCGGTGCACTTTCAAAAAGATAGGGACCAAACCTGCAATTT

novel_mir_85:

ACAAGACACCCATGTAAATCGAGGATGTGTGCATCTTCCAGCTTGTAAAACGTGCTTGAATTACGAGGGATGCAACA

novel_mir_86:

CGCCCGCCGCTGCAACGCGCGGGTACCCTGCTAGTCAATAAATTAATAACGTTAAACAAAACCTAACGTTCAAAATAACTGAATTACATATAGTTTATAATCTATGAAGAACTTCTCTATATACAACGTTGGCTGTAGAATTGGTGTAGTTGTCGTCTTTGTCACAACACAAAATGTTTAGACCTCTCTTGCTTTTTACTCTTGAGACGGTTACATAAAGTTGTCCGTGGGAAAAAAACAGATCTTTTCAAATAAAGCCCAAGCTTAGACAATGTTTGGCCTTGACTTTTGTTTATAGTCATGGCAAAACAAACGGAAATAGGGAATTGTCTCCTATCAAGTTGAAAAAGCATCTTCTTGTCAGATGGGCT

novel_mir_87:

CCTTTTTCGTAGAGTACATTCCAACATAATAGTTATAAAAACAGAACAACCCTTGAGAACAACCAACTATTGTGGTTGATTTAGGAGCAGTTAAAGAAACTTTTTGATGTGGGAAAGTATCATTGTCCACAAATGAAACATAATTGTGTGTATAAAAATATCGTACAAGGATATGTTGTTGTAACTGACTGTTTTGTTGGTATTCAGTGATGAATT

novel_mir_88:

AGTGTTTTGGCTTATTTTGGGACTTATGGGCTTATATAAGTTTATTATGCACAAACTTATATAAGCCCACAAGTTTCAAAATAAGCCGGAAAATCG

novel_mir_89:

GACCGATTAAGCTCTTTGGGTCATTCTAAGCATCTTCTCAATGTTCAATACAGAATGCATCCTTCAATTAGTTTCTTCCCAAATCTGAAGTTTTATCAAAATCAAATCCTAGATGCACAAAATGTGTTATCTAAAAGTTACGGAAAGAGGTATCTTTCAGGACCAATGTTTGGATCCTATTCCTTTATTAATGTCGTTGGGGGAAGAGAAGAAAAAGATGACGATGGAAGAAGCCAAAGAAATATGGTTGAGGTGGCTATTGTGATTAAAATTGTTAAAAATCTCTTTAGAGCATGGAAGGACTCAAAGAAGAAGCTTATTATTGGTG

novel_mir_90:

CATGGGGGTGAGTGTTTCCAATCAGTTGAAGCCTTTCTTGAGAAGGAAATGTAGGCTGATTTGGAAGAGCCCCTCCAT

novel_mir_91:

CTGGATAGATTTACTGACCCGGTCCAAACTGTCACTCACTTCTTCTAAACAGTCTCCAATAGCTGACCTTTCACGGGGTTTTAAACCTTTGATTCTCTTAAGCTTGTTTAAGTAAGCCTGAGTTGACAGGGACCGCTTTAGAGATACTGCTAA

novel_mir_92:

TGGCTCCAAATAAAATTTCATATGCTACCTAAATAGCATATACGAGGCAACTTTTTTGCTTCAGTATGGTACACCTTTAAAAAAAATTAAAAAAAACACACTTCAAAAAGTATTTTTGGTTTGTAACTCGAGAAGCAACAATCCTAGATTTAATTGGGAACGATATGGAAGTTTTAAGGTGTGTGCGCGGTGGGTGAGGGAGGGTACGATAGTGAAGCAAAAGGGTTACCTTGTATAAGCTATTTAGGTAGCATATGAAATTACTGTTTGGAGCC

novel_mir_93:

AGTTAAATGGTTTGGTGTTTTCGGTGTTTCTGGTGTTCCGGTTAATCTCGAGTTAATTGGAGCAAACCAAACAAAGTCCTAAAAGCTCAGAACTCTAAAGCATTCGACCCGAACTCCAAAAACACCCAGAACACTAAAACCAAATAACTG

novel_mir_94:

GATTCGGATTAATTCGGGTCGGGTCAAAGTCGAGTCATGGTTAAAAATTTTATACTCGAACTTGACCTTATTTGAATTTAGATTAGTCGTGTTCGTGTCA

novel_mir_95:

ACTTCCATCACATTTTCTTTGGTTGTCCTCACCCCAAGAATCACTGTAATTAACCCATCGTTTTCCATGTGCTCCTTGGGGTGAGGACAACCAGCGAAAATGTGATGAAGTC

novel_mir_96:

AGTTTGGGAGTAGTTTTGTTCGAGCTCCTATGTGGGAGGTTGGCCTATGATCCAATTTACTCTTCACACAATAGCCTGGGACTTGCGCACACAGCACGACAGAACTTCGAAAACAGAACAGTAAATGAACTTGTAGATCCTAGAATTAAAGAAGGAACTACTGTAACCATCTTTAGATCAGTTAAAGGTCCTAGTCAAGATTCTGTGGATGTGTTTTCAAAGATCGCATATCAGTGTTTGACAGAACCACAAACCAATCGCCCAACAATGGAAGTCATTATCAAGGAACTTGAGAAAGCACACCTCTTTC

novel_mir_97:

TTTGAAACTTTTCGGCTTATATATATAAGCTTATTATGCAATAATAAGCCTGTATAAGCTGAAAGTTTTTTA

novel_mir_98:

TTGTTCAACCACCGTATGTCACCGTCGTTCAACCACATCAAATTAGTGAATTTGGATGAAATTAAACAAGAGGAAGCTGAAATTAGTGAAGGGGATGCTTTGTTTAAATCAGACTATTCAAGTGAAGAGAATGGTGCTTAATGCAGTGGATGTTGCTG

novel_mir_99:

AATATTGATTTTTGATGATGTTTTGTTGTTGTTTGCGGTTTTCGTGATATTGGGTAGATAGGATTGTTTTAGTGTTAAGTAAAGTTGGTTGCGTTAGTTCATATTTAACGCAAACATATATATGTGCACACTCAACATTTTTAGAAGTCGGACAT

novel_mir_100:

ATCCGACTCGAGAACACAGCGTAAGTAGTCGCCCATCACTCATTCTCAAACTCCACCTCTTCATCATTATTTCTTTTTCTTGTTTTCGCGCATTTCGTAAAACAAAATAACAAGTTACATAAAAAAAAATGAGTTTTCACGAAACTGAAGATGGAAGATCGTCGTCAGATCTTCGAAAACCGTTTTTACATACTGGGAGTTGGTATCGAATGGGTTCAAGGCAGTCAAGTATGATGACGTCATCTCAGATGATTAGGGACCGATCTGTCTCTGTTCTTGCTTGTGTCTTGATTGTTGCTC

novel_mir_101:

TTTGGAGTTGAGTATGCGTTTCTCGGTCGCGTATTTCTTTTGCCTTGCGACGGTGGGTATCACATCTTTCGACCCACCTTTGTTGCCTCATACGTGCTGACGGATCCACCCACATTTCGTTA

novel_mir_102:

TTACACGACTTAGCTTACGACATAGACGACATACTTGATGAAATGGTTACCGAAGCTTTGGGGCGTCAGTTAAACCTTGAATCACATGCTATATCGTCTATGTCGCAGGCTAAGTCGTGTTT

novel_mir_103:

TACAATATTTTGTAGCTAATTGGCTACAATATTATGCTTAAAACTGTGGGTCCTGACTCCTTTTGTGGGTCCTGAGTTATTTTTGCGGGTCACTGATTAATTTCTTGCGTATTTTTGGTGGAGGATTGTAGCTAGTTGGCTACAAAAATTGT

novel_mir_104:

TTTGCCTACGTTAAATGATCGTTTTGGGAAAATGTTTCTAGCGAAAAACTACGTTAAAATTAGAAGAAATAAAACATGTACATGGGTACATCACGTGGACGCTGTCACGGCTTTAGCATTGTCACGCGATGGGTCACTTTTGTACTCAGCTTCATGGGACAGAACATTTAAGGTATGGAGAA

novel_mir_105:

AATTCTCCCTTCTTTTTTTTTTTTATCATTTTTTGTGGGGTTGATCGAACCATATATTCATTTTTAAATAAAAGATATGAAGAAACTGGTGTGCGTGTTTTTTTGAATACCTTGGTGTGAAGAAGATTCAGGAAAAAGGGCTTTGGGATTTAT

novel_mir_106:

TCTCAGAAAGGTGTTGAACCTTACAAGGATAGTGAGAAAACAATGATGGTTGATCCTTTCCTTTTCTCTGTTTTCTCATCATCCTTGTAAGTTTTAATCCTCTCTGTCTTCT

novel_mir_107:

GTGTTTATTCCTACAATCCTTCTCATCTTATTCATCATAGAAGAGAAGGATTGTAGGAATAAACACA

novel_mir_108:

TTTTCAACTATTGGTGACGTATCCTTGCATGATCGAGAGAGTGAAGAAATTTGTAAATAGCTTGCCAAAGTTCGACACTCACTCGATCATGCAAGGACACGACACCAATTGTTGAAAAC

novel_mir_109:

AGTTGTCGTTGCATCTGCAGTGCAGCTCCTGATACATCCTCAAGGTTCTTTTTTCTGGTACTCATTGCTGCTAAATGCAACATATTATTTTCGTCTTTATCTGTGATAGGAGTTACCATATCCTTCATTGAGCCTATCTCGTAAAGTAGATTGTAGATACCCTCATGGCGATGTATGACAGCAATGTGAAATATAGTTTGATTATTATCGTTCACCTTCCATATCAGATCAGGATATTGTCGGATGAGCTCAAC

novel_mir_110:

TCTCTCACTTTCTCTGCCGCATCCATCAATTTGAACTTCTTTAAGTGCTCTCATATCAAAAGTGGTTGTGGCAGGGGTGATTAA

novel_mir_111:

GTGATGTAGACCAAATGACAAAGCTTGGAAGCATGGCTTTTTTGTGCACCATGATGGCTAATTTATTGCCTTCTTTAGCAACAATGGGCAGCAAGGAACTATTCACAAACATCGT

novel_mir_112:

TAACAGCCGCACGGTGTATAGCGGCAAATAATTATAAATGCGGCTATTAGGTGTGACAACATGGACTAATCATTTGCCGCTATACATCGTCATTTGCCGGGTTCAAT

novel_mir_113:

TTTAACGGTGAGAACCATCGTGATTGGCACAGAGCTCGTGATTATCTCGTTGGAATTACTCTCACGTAGTAAGTGTCAGAGTAAATCCAACGAGTTGACAGCAGCCAATCGCCTATGATAATCACGAGCTATGTACCAATCCCGGTGTGTTCTCACCGTTACG

novel_mir_114:

TTTAACGGTGAGAACCATCGTGATTGGCACAGAGCTCGTGATTATCTCGTTGGAATTACTCTCACGTAGTAAGTGTCAGAGTAAATCCAACGAGTTGACAGCAGCCAATCGCCTATGATAATCACGAGCTATGTACCAATCCCGGTGTGTTCTCACCGTTACG

novel_mir_115:

TGTGGGCTCATGAGCTTATTTGATTTCGGCTTGTGTTTCATAAGCCGAAAAAAAATAAGCTCATAAGTCACG

novel_mir_116:

TGTGGGCTTATGTGATTTCGGCTTATTTTTCATAAGCTCAACTTTTCTAGCTTAGTCCTAGTTTTTTATTGCTTATATAAGCTTATATTAGCTCATACATCTCAAAATAAGCCAAAAATTTTAGCTTATGAATAAAAAAGCCAAAATCATATAAGC

novel_mir_117:

CCTTAAAGTCTTCGGGGAACATACTACATAGTAAGAATGATTTTTTTTGTTCGCTTTTCAAGGTAGTCATAGCTTAGCTTCAAATGAGCAAATTCTGACCTTATAGCTAAGCTTTGACATACGAAGAGAACTTCAAAGAATGGGAAAAACCACTTTATAACATAGAGAAACATGTAACTTTGGGTATTGATCCAGTTATAAGGTCATAATTTGCTCTTTTCGAGCTAAGCAATAACTACCTCGAAAGCGAACAAAAAATTCAATTTTCTTCCTATGTAGTATGTTTCTCGAAGACTTTTAAC

novel_mir_118:

TGTTTTTGCGCAAGCTGTTGAGCTGCGTCGGCCTGCTCTTTAAGCAACAACCGAACAGTGTTTTCATCCATGCTCATGAGAACAATGAAAGCACCAATCGATACAACCTCAACGGGTCGTATCAATTTCTTTGTGCCTCACCGGTGCAGTCGACACCCTTGTACTTCGCCTTGGTTCAACAACAGTCCTTGGCGTATCATTC

novel_mir_119:

TTACACGACTTAGCTTACGACATAGACGACATACTTAATGAAATGGTTACCGAAGCTTTGGGGCGTCAGTTAAACCTTGAATCACATGCTATATCGTCTATGTCGCAGGCTAAATCGTGTTT

novel_mir_120:

AAATGATGCTCTTTCTATTTTTCCACGGCTTTCTTGAACTGCATGAGTTTTGTCTAATCAGATCTGTGATTATATATTTATAATCGTTGCGGTTCAAGCAAGCTGTGGGAAGATAGAATGAGGATCAACACCA

novel_mir_121:

ATGAAGTTGATTGGTAACCCCGACTAGGGGTGTTAGTCGGGTCGGGTTGGTCGGGTTCGG

novel_mir_122:

TGCACTTGTCAAAGGTTGGATTGTTGGCTTCAGGGGCGGATCTACATACGATCCACCCGTGGCACGTGCC

novel_mir_123:

AGACTAAGAGTGTGAACCACAAGTCGGATATGTACTCGTTTGGGATAGTTTTGATTCAATTACTGTGTGGCAAGGAATCAAATCCTTTTAATTATTTGGCTGTGCGATTCATTACCCATTATCGAGAGAAAAAACTCGATAATATAGTTA

novel_mir_124:

TTTGTTCTAATTTCACTTGTAGATGGAGAGTCATTGATTGTCCGATATGTTCAAACCTGTCGCATCTCTTGATATGGAAGTGAGGTTACTTGAAATTTCTAGAGAACACA

novel_mir_125:

TGAGGGAATTGTTGTAATAGATGTTTTGGAACAAATTAAACTGATGTTTGTTCCGAAAACGTCTGTTACAACTTTTTCCTTG

novel_mir_126:

TGAATATAAAGTGGACAATTATTATAGGAAGGAGACAGTATCATCTTCTATTATTAAACTAGTAGCAGCTGTTAGCAGTCGTTGTTTTATAACATAGTTGATAACTTTATATGAT

novel_mir_127:

TTTTGTTTAGTAGCCAAGGATGACTTGCCAGCTCTAATACGAGTGATCTTTATATATCTCTCGGTGTTTGGATCATTTGATTGGTTTCTGGCAGTCAACCTGGCTATCTTGACAAGC

novel_mir_128:

CGCCTCACTGTCCATGAAAGAGCTTGACGGGGTGCTATATTCGCGTTAACTTTTTCAGAAAGCGTGAGGTT

novel_mir_129:

GAGTCCCCCCCTTCTTGTCTCAACCAATCAAAAGATAGTTTAGTGGCCGGGGATGATATTTGATTGGTTGAAAAGTGTAGTGGTCAC

novel_mir_130:

GTTTAACCACTCTTATATTCAGCGATAAAACAATTATACTGTGGATATTGAGAAACTAAAACGAAGTTGTACCATCTCAAGATGAAGAATGGTCCAAGTTCGTTTTAGCTTCTCCATGTCCACAATTTAATTGTTTTGTCGATGAATATAATAGTGGCTGAACCTATTGTCTCACA

novel_mir_131:

GCTGTAGCCAATGATTTTTGCGTAACTTATTTGTATTTTATTTCTTAGTAAAATTTTGGAGTATAAATTACGCGAAAATCGTTGGTTATCAGAAGATATTGGAGCAAAGA

novel_mir_132:

AGGTTTTGATAACGAATTGTTAATTGGAAAGGTTTACAAGGTTGTTGTTTTAGATTATGTCCATCTAGTGAAGATAGGCATTAATTCTTCTTCTTGTGGTTTAGACTACTTTCATAATAGTGTTGTCATTCAAAACGG

novel_mir_133:

ATGTCTACCGTTTAAGGTGGCAATGAGGATGTGTACTCTGTACCTGTGGCCACAAAGTATCCATCCTCATTTCCCACCTTGAACAGCAACCAT

novel_mir_134:

AAATGATAGTCGACATATAACTATCTAAGTCAACATTAATTTAACGTTTTAAAGTAAAAAATAAAAATAAGAAATCCACGTATTGTTTTTTACCGGTGTAGTCGGGTGCCTGTAAAGCCACATAAGCAAACGTATGATGATGAAAAAAAAAATTATAAAGTTGGATGGGTATTGTTGTATTATTTTTT

novel_mir_135:

TCGTTTCTATTCCAATTGTCTACTACTTAAACAACTATAAGTAGTAAGTTTATACTACTGCTTATTCATACTATGTATTATACGTAGTAGTATGAATTTACCACTTCTAGTCGTTTAAGTAGTGGACAATTGGAAAAGAAACAA

novel_mir_136:

GTATTTGTGATAAGGATTTTGTTATATCTTGTTTCCGTGTTTGTTTTTAAGTTTGTGATGTGATATGACATCATTTTACAACACCGGAGCGGGGTATGGGGGAGGTATGATGTAGAT

novel_mir_137:

CATTGCCTTCCTCCCACATCTGTTCGAAAAGTTTCATATACATACATACTAGTATGAAGCCTTTTGAACAAATCTGGGAGAAAGGTAGTC

novel_mir_138:

TCATTAATCCGATTTAGGGTTTGTAGCTGTTGTTTCTTGATCTGCTCCTGCTCACAGTTAATACTACAATCCCTAATTTGTAACACATA

novel_mir_139:

ACTCTGACAATCTGTCTTATGAATTTGGCCTCGTGTCCATTAGCTAATACCATTCCTGTCAAACTTCCTGCTTTTCTAAGAGAAGCCCTCCATGCATTCACCTTCTCCGACCATTGAATCTTCTTCTGATGGTCTGGCTCTGTCTCGATTATATCATCATACCGAGAAAACGCCTTTTCAAAGCTTCCTGTTTGATTTCGGACATCAGATGGCTCAATATTGTAGAATACAGGAACAACTTCATGCTTAGAGGAAAGCCTCTCGTTTTCTTCTATAATCGTCAAAACCTCATCAAGGCACCACTTGGAAGATG

novel_mir_140:

TTGCAATACCAATGACTATCTAGAGTCATTAGATTCTCACGTGAGACAAGTGCAACACAAGCTTTTGGAAGCACAAGTCTCATGTGAACTAATGACTCAAGATATGCATTGATGTTGCAA

novel_mir_141:

TCAATGCATATCTTGAGTCATTAGTTCACATGAGACTTGTGCTTCCAAAAGCTTGTGTTGCACTTGTCTCACGTGAGAATCTAATGACTCTAGATAGTCATTG

novel_mir_142:

AGCTACAGCGAGAGCTGTTTTTGATCTTCTATCTGCTGATTTCGAAGCTAACAGCTTTGTTGAGAA

novel_mir_143:

GAGACAGGTTATCGGGTCGGGTTGAATCGGGTTTTGAATTTTTACAAAGTGCCCTACAACCTGACCCGATTAAGAAGGGTTCG

novel_mir_144:

AGTCATATTGTTTAAGTCTCAATTATCATCATCTATGATATTTTTCATCACCAGTTAAAGGCTTTTTTTCAGATGGATGAAGATGATGACCATTGAAGACCTAAAAGTTGATG

novel_mir_145:

ATCCATTTTAATCAGTCGTTTGGTTTCAGTTCTGTCATCGAAACCACCTGTACAACCACATCTATTCCAACCATAAGTTTCTACCACTCATACCACAAGATCTATTACCACAAAAAGCAGATCTACAACTCAAACCACCGAATCTGCTATACAAACCACAAGATCTACCACCCAAACCAGACACAACTGAAACCAAACGATTGATTAAAATGGTG

novel_mir_146:

ACCGATGGCTTAAATGGCAATCAAGACTCCCATAAGATGTGTGTTCATATAGATGTCTAATAGTAACAGTGGCTGATTGTAATTTGATCTCTTTCCATTGGA

novel_mir_147:

ACTTAGGGAGTGTTTGTTTCGGCTTATATAGCAGGCTTATGGCTTATTTGGTTTTGAGCTTATGTTTCATTACTTTTTGTGGCTCATTTTGCAACCTTTTGGCTTATATAAGATTATTATGACATAATAAATATATGAAAGCCGAACAAACACCCCATTAA

novel_mir_148:

CATTATCACCATTGTATTTGCAGCAGCTATTACAGTGCCCGGTGGAAATAATCAAGAATCAGGGGTACCTATTTTCACTAACAACATTGCTTTTAGAGTATTTGCTATATCAGATGCAATATCACTATTC

novel_mir_149:

TGGAGGAACGTGTGTTATTCGTGGGTGTGTTCCTAAAAAGATTTTGGTCTATGGAGCAACCTTCGGGCCTGAAATTCAGGACGCAAGGGAGTATGGATGGCAAGTGAGTGAGAATGTTGATTTTGACTGGAAAAAGCTACTACAGAAGAAGACCGAGGAAATAGTGAGGCTAAATGGGGTATACAAGCGCTTACTTTCAAATGCGGGAGTCAAACTGTTTGAAGGGGAGGGGAGGATTGTCGGCCCTAATGAAGTGGAGGTGATACAGTTGGATGGCACTAAACTGTCC

novel_mir_150:

TTTCAAGACACGAATCTGAAACTAAATGACGATTTTGCTAGACCAAAGTTTCATGCTGCTGCTGCTATGGAATGGTTAGGCATACGGTTGGATATGCTGTCGTCTTTTACTTTTGTTGTGTTTCTAATTTTCTTGATA

novel_mir_151:

TAAGTATTTCATTCTCGATGCAACTTCTCTCACATTGAAAAATGTATCGCTGAAACGATCCATGCCCAGCGAAACAAGTTACATTGTGAGAGAAGCAGCATTGAGAGTGAAATATTT

novel_mir_152:

GACCCCATAGGGGGGTTCTAATAGTACAACCCGCGGGGTTTTGTGGTGGCGGATCCTGCGTATGTGACCTCGGGTGGTGGTGAGATCCGGCAGTGGAGGGGTACGGTGGTGAGGTTGTGGTTTTTTTTATGGGTTGTACTATTAGAACCCCCTATAGGGT

novel_mir_153:

AACAAGTTGCTGATACATGTCTGGTGTCAATCTTCCAGTGCTTGTGCAGGATCCTGACTCAGACACGGTGCAAGTGTA

novel_mir_154:

GAAGAAAAAATGGAACTGCATGATCAAGGGTTAGAAAAGTACCAAGACATAGTTAGGGCTGCGTCACAGTTTCTGTTCTACAAATCCCCTGAGGAACTTAAAGAGCTTCTCTCCATAGGTTTCCACATTAACAATCACAAAACGTGGTTTTCACTCAATGAAAATGGAGAACACAGTGAAATGTTATCTATAGCGGATTGTTTGATTCCACATGACGGTTACAGTTTATGTTATACTGAGCTTTATTCAAGGTGACTTATCCTCCTCCTCCACCTCCTCATCCCTAATAATATTTCATAAGTATTATTCTATGCTTCTTCTTCATGCAGATTTCCACGCTTTTTAAA

novel_mir_155:

GAATTTGAGATTTGGACATGCAACGGGCAGGTGAAGTTCTTCAAAATGAGCACAATACGAAAGAGATAATGTCTCGAGATTCGGAGTCA

novel_mir_156:

CACAGATGTAAGCAGGGACGGAACCAGGGCACAATCTGACCAGGCGCATTTACGAAAAAGTATACAAAACGAGGAACACAATTTTTTTCAACCAGGTGCATTTAAGAAAATTCGAGATTTTTTACCAAAATTAGCACTACACTGCAAAACCCTGGTTCCGTCCCTGGTCACAAATGTT

novel_mir_157:

TCTATCCTACTTGTCACAGCTATTGTCGGCATTGTAGTTGGAGTTAACTCCAAAACTTCCAACAACAATTCCGACAATAGCTCTGACAACATTAAAGCA

novel_mir_158:

AAAAGATACATCAGAGGACGTACATGAAGAAGAAAATATTGACCAGGTGTTTTTTAGTCGTTTACAACGAAAACCTGCTGATAAGCCTAAGAAGAAGAAGGCGAAAGTTGATTTTTTTGAGACTTTGGTTAAGGAGTACAGTAATGAAAATGAGAAGTGCAAAAGTGACGAGAATGGGTTAGAGAAGCGGCACATTAACAAAGATTCTTCAGTAGTTCGTGATAGAAAAGTT

novel_mir_159:

AGCCTTTGTAAGGATATATATGCAATTCCCCCATTAATAATTAAGTATTACTTTCGCTATGGGGATTGATAGCAGCTAAATCCTCCATAACATAGGTTG

novel_mir_160:

CAAGGTCAAGGGTCTTCAACTTTGTATGACTGAGGTCGATCTCTTTGAGCTTTGTACTTTCAGCGGGCATTTGAAGTTCTACCAAT

novel_mir_161:

ATATATCTCACCAACCTTTCATTAAGTCAAGATTGCCATTGCCAGGTATATTGCAGAGTTTTAAGAGACTGAAGAAGCCATTTTAGAGAAAGAAGCCAGCTGGGTATTAAATGTAAGTTTAAAGAAGCCACCTGAAAGATGCACCAAATTTTGGAAGGATCAAAGATATGTTTGGCTTGAGAAAGGTTTAGTGGCTGCTAT

novel_mir_162:

TGGAGGCGCGTGACCGGGATTTAAATGACTTGGTGAGGTGTTTTGGTCGAGAGAAAAGTAGCTATCCTGTGGTGGTGTTTAAATTTCCCGGAAAAATTTTAGGTCGCCGGCGGCTGTAACGTGGAGGCGCGTGACCATAATATCCGTTAGCTGTAGCCTTCAACTCCCACCCGACCAGATCGGCTAATTCTTTCATAGCGTTTCT

novel_mir_163:

TTCTTGAAGGCTCCATCTCTTGCGTTCATCAGGAGCTCTCACTGTTGATGTAGATTGGGCCCACTTTAAGAC

novel_mir_164:

TTTCAGAATTTGATCATGGGAAGCAAAAGTAGTGAGTGGGGACATAGCCCATGTCTGAGCAAGAGGTAATTGGTGAGTGTTGCACAGAAATTTCAGTATATTGTGGATCTTTTCCAACTCATTTTGCCTATGCGTGGGGCAGTCAACTGCTTGTGTGGTTATTAGATTTGCTGTCTTCAGGGTGTTTTGAAC

novel_mir_165:

CTCTTAAAAGAAGACTCTGAAAAGTTATCAACAGATATGGGTGTTTGTGATGACTTATTCAATGCTTGTATTAAAAA

novel_mir_166:

AGCTGAAAAGTTTCAAAATAAGCCACAAAAAGCAAGCTAATGAAACATAAGCCAAAAACCACATAAGTCATAAGCCCCCAATATAAGAATAGCTATAGCCTTTCATTTGACACAACAGACACAAAGTATTCTTCGGGGGTGTTTGTTTAAGCTTGTACGTGGCTTATGAGCTTATTTATTTTCCGGCTTATTTTGAGACTTATGC

novel_mir_167:

ATTGGTAAACCCCATCACAAAAATGATTCAGTGCCCATGTGTTCGTTGTGGTAACGCTCCAACTAAATTGGGTACGCTCAAAACTCTTGAGTTACACATATCTAAATACGGGTTTGACACACTTTATACCCCTTGGAAATATCACGGTGAACCACTTCCTCCACCCGTAGTACATAACATACCACAACCACCTTCACCTGAACACATGAACATGGATGCATTCTTCCAA

novel_mir_168:

GAAATGTTAGTAGACAGTTGGCATATTCTTCTGAGGATGAGAGGCCCAAATGACGAGGCTTGTGATCCCATTGCCCCACTCGAGTTGCCACATTCTTTGCATGCCTTCAATCGTGTGTCTGATACAGATGAACTTAATGTGTCAGGCTACGACACATTTCG

novel_mir_169:

AATTTGAATATCAGAATAGCGGATATAGTCATGATTCAATGGGTCAGGTCCACTTATTTTTCTTTTATTGATTTCTCATTTTTACAATGGATTTCATTGGCCTAATTGAAAATAGGAATTTTTAGTGATTCAACAGATGACTTATCATTATTCGTGATAACTATAAC

novel_mir_170:

GCTTATGTGATTTCGGCTTATGTAATATAAGCCTCAATTTTATGTCTTATTTTGAGATTTATGGACTTATATAAGATTGTTGTAACACAACAATGTTATATAAGCCAAAAAGTGTCAATATAAGCCACAAAATTAAGGCTTATGTTATATAAGCCGAAACACATATAAGCT

novel_mir_171:

TCGGGACAATCTCATCAATACTTGGACGTTGCGATCGTTCTTCGTTTAAGCAATCATGCGCTGTTTCCACGAAAAGTTTGAATGATTGCGGATCCATTTGCTTCCAC

novel_mir_172:

GAGGCCATTCAGGAGAAGGTAACAAAAACAATTACGTCCGCGTTTACCTCTATGTTCACAGGTATTGGTGATACCATGGACACGTATTATTAGATGAGGAATACAATATATTAGGGGTTTTATTTTGGTATGCAAATTGGTGGTATTCAGAGTGCGGTTTAATAGTACTTTTCTGCTACCGGTTTCATTTTGACATCATGTTCATTTTGACATCTTGCTCTTTGGCTGA

novel_mir_173:

TGTTTTAGGCAATACAAGTCGTATTGAGGAGAGGGTTTTGCGGATCATCTACAATAACACCTGCTTTCATAAATTCTGCTCAATACGAACTTTATCGTTTGAAAC

novel_mir_174:

GAAGATTATTGTCCTCCTGTATTTGAACAAGAAATGGGAGATTTAAACCGAAAAAGAACAAGATCAACAACAGGAACAGGTCTAAACAAGTACGACTTATTGAGAACCACAACCACTCAAGAAGAAGAACAACCTTTGGTTAGTCAACAAAATGTCAAAGTTCGAGTTCGGGGTTCGACCAATCTT

novel_mir_175:

TGTGGTTTGGATTACTTGCATAACCATGTTGGAGCTCATCAGACCATAATCCATAGAGATATAAAAAGCTCAAATATTTTAATCGATGAGAATTGGGCTGGTAAAATTTCTGATCTTGGATTGTCAAAGTTAAATGCAACAGGTTTGGGTATGACTCTCATTGTTTCAAATGGTTGTGGGACACCAGGTTAT

novel_mir_176:

GGCGGCATCGTAGCAATCCTTGTTTATTAAAGGTGTTAGGAGTACTTTTGCTGGAAACATGCGAACCCGGGGCTCATTTGAACGAATAATGGAAGAAAAAGACAAGTTTCAAATGTGTGACGAGCAAGTGTGACGCTGTCGCCA

novel_mir_177:

TTGTGTTCTTGATGTCCTCCTCTTGATGCTTATCAACTGGTTGGTATTGAAAATGAAATGGTTGGTATCAAATGTTGGTTAACTTCACTGATGAAGTTGGTTGGGCCGACAAATGGTTTAAAGCAAAAGGAGGTGGGACTAGCTTTTTTTTT

novel_mir_178:

AATTGATATTCACACCATCAAAATTGATACACACATCACAATATCACATCATTAATGCACATGTTACAACATGATATTTGTATAATTATGGTGTGTGCATCAATTTTGGTGGTGTGAGTATCACTC

novel_mir_179:

ATTGAGGTGTTTAGTTTGATTTGTGCGGCTCCAATAGATTCGTAAAGTGAGATCGATTGAGGCGTTTAGCTTGATTTGCGCGGCTGCAATCGATTCGTGAAGTGAGATCGATTGCTGAGGTAGTGATTTCTTCACCTTTTGATTCTCATTTTGATTTGCGCGGCGCATTTGTATAGTTATGTACTGATTGTATTGTTTTTGTGGAAGTATTTGTGCTAGAAGTTGTTGTTTGGTTCATGCTTATAATTTGATAATGAATGAGCTGTTTATGAATTAGTTTGAGCACTCAGGTGGGAGGACAGACTTAAGCTTGACATGTCGGA

novel_mir_180:

TAAAAGCGTTTGGTGACATAGTTCTAACCAGTGGTACCCCTTGTTGTCTGAGAAGCTCCGCAACCGAATGGTTGTGGGTTCAAGTCCTGCTTAGGACATGTGAGATAGTTGTAGTGT

novel_mir_181:

GAGCTTATGTTTCATAAGCTCACTTTTTGTGGCTTATTTTGACACTTTTTGGCTTATGTAAGTTTATTATGTTATAATGGAGTTATATAAGCCGGAAAGTGTGAAGCCACAAAATGTGAGCTTTTGAAACGTAAGCCCA

novel_mir_182:

CCCAGGATCAAAGTTCATAACTGGAGATGCGGCGTTTGAAGGAAAACATTCCTTTCCCGGTAATCCATTTTTGGACCTCGTATGCTCCTGTGTGTTACCGCCACCACCGAATATTAGATTAGGAACATCATAGGCCCCTTCCATAACATTGGTGTGGCCTGTTGGGATATTGCCACCTAATTCTTTATCCAAACAAGATGATGAGTCGTGACCGGTTCCCTCTGCTGCTGCTGCAATTGAAGCAGCTCCAATTATGATATTGTTGTGTTTGCCCGCTTTGGAAATTGTTCTACGTTGTAATGCCAGTTGTCTTAGCCTGGA

novel_mir_183:

CAGTGAAGAAGATATCTAGATCTACTTTATGATAAGGTTTTTCCCCGGCGACAATCCCCGGTGACATGTCGCTGGGGAAAGGTATAGTTTTTGTCG

novel_mir_184:

GAAAATGTCAACAAGCAGTTTCGGATTTTAGTTCATGCATGGAAGAAAAGCTGTGAAGAGAATAATTTAGATCAATTTATCTTTCATTGTCTGAAGCAACAAATGGATTCAAGTTCTTTGAGATTGTTTTCTGACATTGCA

novel_mir_185:

ATCACATTTATGAGCGAGGATTCGATTCAATAGACAAATAATTGAGATCCGTAGCTTTGTAAAAAGAATCTTTGTCTACTGAATCGAAACCCGTTCACATGCTCACA

novel_mir_186:

AGAACTTAAAACGTCATTGAGTGCAGCGTTGATGAATCGACTCAAAGCATGGAGTTTCGTCAACACTGTACTCAATTATGTTTTTGCTTC

novel_mir_187:

TTGGCCATGGTTCCTTTACATACTTGTGCAGATATTCCTTCACACCAGTCATGCTTCTTGAGCATCCACTGTCTAGATGCCAGATAGGCTCCCTGGCATGCCTCTGCACAAGTAGTAAACTGAATTAGTTTCTC

novel_mir_188:

TGTTTTTTTACGTTTTCGTCTGTTATTTTAGGTTTTCGTCCGTATCTTTATGTTTTTGTCCCTTTTGTTCAGTTTGACAGACGAATACGTAAAAAAAAA

novel_mir_189:

TCATTAATCCGATTTAGGGTTTGTAGCTGTTGTTTCTTGATCTGCTCCTGCTCACAGTTAATACTACAATCCCTAATTTGTAACACATA

novel_mir_190:

AAGCCAAATTCTCGATACGCCATTGAGCTAGACGCGACGTAGTCTCAACACGATACGCGTTATCAGTCATCCTAGTAAAAGCAAGAGCCGACGTAATCTCATGGAAACAATCAAAGATTCCTTCCAACAACAACTCCCCGTGTTTTCCCAGGTTGTCGTTTAAGGTTCGTGTTCTTGTCCTTGTTCTTGTACTTCATATAAAAAACTTGCTAGCTAGCTAGGTGTGCATGATGATCTTGCCTCCAAAAACTTCGGGTGGTCGGGTGGTGGCGTGTCGGGTGGTTGCTGCTGTGTGGTGGCGGCGGTTGGGTGGTGTTGGCGG

novel_mir_191:

CCACCTTTTGTACTCGTTAAGGTGGATCCCTTTGGAGAGAAGCACTTTCAGTTCCGCAAGGGATCTGTAGGCAAGAGAAGGTG

novel_mir_192:

AGGATGCACTTTGAGCTTGTCCAGTTCACTAACCCACTGATCCATAGTTTTGCCGTATAAAAAAAGACCCAAATGTTTGAGGGCCAGAGGGTGACCTTGCACATAGTTCACAACTTTCTGAGAAACTTCTTCAAATTCTATAGAAGAATCGTTTTCTTTAAGGGCATAGGAACGGAAGAGCTCAAGAGATCGA

novel_mir_193:

TGAAATGTTAAAGCTGATGTGGCTGACGTTATTTTATAACTTGAGTTACTGATGTAATTGCTCTTAGTGACTGAAATTTTGCTCAATTTTATCTAGCGATCTAACTGTATCAAAGCTGGGCCTGGCCTTCACACGAAAAGCAAAAAAAACATACACATAGATACCGATTACAATTAACTGAACAACCCAACGTATGAAATTCCAAACTGTTTATCAATTCTATTATCCACCACACTCAGCTACTAAACTGTCAA

novel_mir_194:

CGAGACAGGTTATCGGGTCGGGTTGAATCGGGTTTTGAATTTTTACAAAGTGCCCTACAACCTGACCCGATTAAGAAGGGTTCGG

novel_mir_195:

CTTGTTTTGCTTGGGGCTTTGTGGGTTTAGGTAATTTGGGCGGGCTTGCTTCTATGACGTCTCGTTCTTTGCCTTAGTTTAGGCCCCGTGCGTTGATGTG

novel_mir_196:

CGGAGCTACGTTGACAGCAGTGGGACCAACTACTAGTGGAGACACAAATTATATAGGAAAAAAAAATCAGATTCAAAGAAGATTGGTCGAGTGCCCTCAGCCTGCTCTC

novel_mir_197:

CAGTTTAATGAAGTTCTGCTGAATGAGATGGATAAGATCAGAAAGGCTTGTATTTCATTGGAGGAGAATTACATGCCTCCTGT

novel_mir_198:

CCCATATCTTTCATAACTTTCTATATCTTGTCAAACCTCTTTTACTTCCTCTCTTATCTTTTGTCTTTCAAACACGCTTGTTTTATTTAAGATTTTGGTCAAGTGTAGATGGTTATGGAGTCTGG

novel_mir_199:

TTATTTGGTTTTCGGCTTATGCTTCATAAGCTCACTTTTTGTGTCTCATTTTGAAAATTTTCAGCTTATATAAGCTTATCATGTCATGATAAGCTTATAGAAGCCGAAAAGTTTTAAAATAAGCCAGAAAAAATGAGCTTATGAAATATAAGCCGAAAACCAAATAAG

novel_mir_200:

TGCAATATCAAGGCATATTTGTAGACGTTGAGCCCAACTTAGTCTCTTCGTGTTATCAATTTTTTCAAAATAATCACTAAGGCTTCCGTTTGATATATACTCGTAAACGAGTATCATCTCATCACCTTCATCACAAAATCCTAATAGAGAGACTACATTTTGATGCTTACACTTACTAAGCATTTCAATTTCTGCAAGGAACCCTTGTTCACCTTGCT

novel_mir_201:

GCATTGCGCGGGTACCCTTCTAGTATAAACAAAGTGTATAAGATACACAGGATTTAATAAAGAAAACCGAAGAATATATTATATACTCACATTTCTGCAAGATCAGCAGTATTATGTCTCCTTGAATCCGCAATGACAATCTGAAATTCCATTAGTTGGTATGTTCTCAAACTTTGTGATTTCAGAAACTGAAGCGTCACTGGTAAGCC

novel_mir_202:

CTTTCTGGCCTAGCGTCCTGGTCCGAATATAACGGCCAAGCTAGAAATGTACAGTAAGAAATTCTTATGATTTATAAGATGTTGTTGGTATCATTTGCTTTTGGCTTTAGAACATTAAAGTCTGCTTATTGCCGGGTGTTTTCGACTGACAAGAGACGTAAAAAAAGGGG

novel_mir_203:

GAGTGCCCCCTTGCCCATATTGACGATCTAAGTGCTTAAAAACGGCAATCCTTCGCCCCCCTGGTAAAGAGACTTCTGCTCTGTATGTCCTTCCATATGCACCCATTGCAATTAAAAAGTTGCTAGAAAAGTTGTGAGTCGACAATATTATATCTTCCAAAGGAATTTTGAGGTGTTGAAACTCTTCCATGGGATTGTAGGACACTGGCACCC

novel_mir_204:

GGTCGTTAACGAACTCGAGGCTGCAATTCATCAACAAAACTCTTCTCAAAACACACCTTACGTATCTAGAAAGATGCCAGTGTCCTACAATCCCTTGGAAGAGCTTCAATACCTCAAAATTCCTTTGAAAGATATAATATGGGCCACTGAAAACTTTTCTCACAGCTTTGCAATCGAATGGGATGATTTTGGAACGACATACAGAGTAGAAGTCTCTCTACAAGGGGGGCGAAGAATTGCCGCTTTGAAGCGCTTAGATCGACA

novel_mir_205:

TGCCATAAGGTCCATGATGTAGAATATAACTGTAAACCTTTTCGCTTCGGTTCAAGACATTCAATTGATATATACCCTGCCCGTCTTTTTTGGAAATATATAATAGCAGAAAATATGTGGTCAGCACTTCTATTGCTTCAGCGGTGTTGTTTTCCACAGCTAAACAAAATGGAGAGCCATAAAGACGTTTAATATCACTTTGTGTATATGTTCTATCAGCTTCCTCGCATATACGCGTAAGTAGCTTAATAGTTGTATTGTGCTTTACTTTATCATC

novel_mir_206:

ACCTTGTATTTTCATGGGTTTCAGATGGATGAAAATTCTGCATCTCATCCAAGACCCTTGAGTCCACCAAACA

novel_mir_207:

GCAAGACCTGTTATGATTCCCGGGATGCCCAAAGAGCAAGTGAGTTTGGCAGAATGGGGGAAATTGTGTTATCGGAAAGGGACCTTGAATAAGATAGTCGATCCAAAACTGAGGGGTATGATTGCCCCCGAGTGCTTAAGGCAGTTCGGGATAGTAGCAGTAAGTTG

novel_mir_208:

GATTGCATATGAAGATAGAATAAGCTTTGCCGTATGGATCTTATCCATCTCATTCTCTCCTTTTTTGACATGAGATATGTTAATCATACTTGTACAGTGTCTTCAAGGATGTTGCGGTGAGAATGTGCGTGGTTTACAAATTTAGCAATGTGAAACAACTTGCAAGTCCAAATAAGGTTTCATTTCCGAGACACTGCACTAGTTCGAATAACATATATCATCTCAAGGACCGGGAAAATGGGATGGATAATATCCATGCGGCAGATTACTTATTCTATCTTCTTGTACAAT

novel_mir_209:

TCAAGTCGAAATAGACATGTCTTCCTTTCCACGTATGCTACAATACGTCCATTGTACTTTTTTAAATTGTAGCATACGTGGAAAAGAAGACATGTCCATTTTAACTTGG

novel_mir_210:

TCTAAGTGATGCTAGTCTCACGTGGAGCCAACGTCTCAAAATATGCGTTGGGGTTGCACGTGGACTTAGCTACTTACA

novel_mir_211:

TCCTCGGTCTGGTCCGGTCCGGACCAAAACCGATGAATTTGGGCTAGTTTGACCTTGTTGGACCGGACCGACCACATTCGGTCTCGGTCTCGGTCCCGGTTTTTACCTTAATTCGGTCTCGGTCTTGTCGGTCTCGGTCCGGTCCGGTCCGACCAAAAA

novel_mir_212:

TTTTGGCTTATTTTGAAACTTTTCTGACACTTTTCGACTTATATAAGCTTATATAAGCCGAAAAAGTTTCAAATAAAGCCACAA

novel_mir_213:

GTGGGCTTGTCGTGCTTCGCCGTCGTCTCACGTGGTGTCTCGCCGTGGCTCGCCGCCACACCGGCGGAGCTCGCCGTCGACGAGCTCTTCGTCTGCACGACGAAGGTGATAGTCTGACACCTTTTTTTT

novel_mir_214:

CTCTGGTTATCTAGGATCGATCGATAAGGGTTCTTGGGTCTTCGTCTTCGTTGGTTTTGGGTGTAGGAGA

novel_mir_215:

TCACGACTCACGACATAGATGGTGGTTGTGGTCTCAGGTAGCGACTGTGACCGCGATCATGGATATGGCTTCAAGTTTTGG

novel_mir_216:

CGAAGAATCTAGGTGACAGTAGTTTGACAGGCAAAAGTACTGCTGTCGTTCCTGCTCACATGGGTTCTGGC

novel_mir_217:

TCATCTCATACGGCTCCTCCCTTATATGCATAATGATAAAATTATAAAGAAGACGAAAATCTTCTCATTACATTATGAACTGAGTAGGGCTAGTGTTTTTACA

novel_mir_218:

ATACCAAAACTATGATTTGATATTGTATTCATCAGATCACCGCCTCTCTTCTAGACACACACACAAAACTATGTTTGATATTGCATTCATCGGGTTTTAATTTGTAGGTATCAACCTTAATTACTTTTCGTCTGGGTATTTGGTAATACCTAATTTGTGCTCGGAGAGTGACGGTGATCTGATGGAGGGTA

novel_mir_219:

TGCATCAGTTCGGTTATCGGGTTGTTCAGTTTATTCAGTTAGGTTATTTTTTATTTTTTTTGGTTAAACTGAAACCTAAACCGAAC

novel_mir_220:

ATTAATTTTAGGATACAAAACGGTGACATGTTATGTCGTTTAGGGCTAAGTGTTTTTTTTGGGCTTCTTCGCTTCGTTTGGCCAAAAAGCTGCTGCAGTTACCGCCTTTGATATATCTAGAAGATTA

novel_mir_221:

TTCTTGAAGGTAATTGCATATGTTGTCGGATTTATACCTTTCTTATGGAAGACACTGGCTTTGTCTAGAACAATTGGTAGTCCAGCCTGTAAACATGCACGCATCTTCTTGTACCAAGGACGCCACTCTACTTCAGGCTTATAGCCACTGTCGTAAAATGTTACAACCCCCGTCCTAATATGGAATCGAGCCAGAAACCAATGTTTTTCCTTTTCATTTATAGGCATATAAACCTGTTCAACATCAGCCCATTGGTGTTTGTACTTTTCCCCATTAACATACCAAATGGGGATGGAGTCTTGTAATAGCAGCTGAACAAAGTATGCAGTTACCATACCCCA

novel_mir_222:

TGTTTTTTCATCACGGGTTTATTGTGGTGGTGGTGGTGGCAACGATTGTGTTTGTTGTGCTTGTTGGAGTGGTGGTGGATTTGTTGAAGATTATGAAATTGTTAGTTAAGTAGTTGTTGTTGTTGAAATAGTTGTTGATGAAATAGTTGTTCGGGTGTGTATGACGGCATATAGTATTGGATACCAAAGGAAAGAAAATTGGGATGTTGTAAGGAAGACATTGAGTGTCCGGACAAAGGCATAGGAGGGTTTGACGAGGATGATTGTTGCGACATATTGAAAAGAT

novel_mir_223:

AGGATTAAAACTTACAAGGATGATGAGAAAACAGAGAAAAGGAAAGGATCAACCATCATTGTTTTCTCACTATCCTTGTAAGGTTCAACACCTT

novel_mir_224:

GCTTAGATAAGCCAATAAATCTCAAAATAAGCCGAAAAAACATATATGCAAATCTAGGGGGTGTTTGGCTAAGCTTATTTACCATAAGCCGATTTGGGCTTATTTTTAATAAGCTCCGATTTTGTGGCTTATTTTGAGACTTGTCGGTTTATTTAAG

novel_mir_225:

GTGTCACCACTTGAGTGGTGCCACGTGCCATCGGGTCGGGTCAGGTCAGTGAATGGGTCGGGTCGGGTAGGTTCGATCAACGGATCGGGTTGTGACGTGGACCAATGAGGTGGTGCCA

novel_mir_226:

AAACGCCTATTCATTGGACAAGAAGGAGAAGAAGGGTTTTTCACAGAAATTGAAACGCTTACTAACTGTAAGCATCCGAATATAGTTTCTCTTCTTGGCTTTTGTGAAGAAAGGCACGA

novel_mir_227:

CTCAAATGCTTCGAATGGATAGTGAGAAGATCAAATAAATATAAATCTTTAGATTGGCAACAATGATTACGGGACCCTCGGTACATTCGGCTACACTGACTTCTAGTTTATTTTGTTTTTGCTACTGTATCGTCGGTGCATAGTAT

novel_mir_228:

ATAAGGTGGAATGTTAAAGATGATGTGGCGCTAACGTGAACGGTGTTATTTCATAACTTGGGTTACCATTGTACATGCTCTAAGGGTAAAACTGTCATTTTGTAAGAAAACATCATAATAACACCGATCTCAATGCATGTACTAAGTATGTTTTTGTCGGTGAACAATTATTTTGAGACGTGTGACACCGTCA

novel_mir_229:

CTTTTTGTGACTTATTTTGAAACTTTTCGGCTTGGCTCACTGTACGAAACATAAGACGAAAACGAAATAAGGCATAACGGGG

novel_mir_230:

GAAAGCTGACGTGGCAGGAAAAATAAAGGGGATTGATTGGGGCTCCTTACTGGGGGCAGCCTAAGAGTGTAATTAAGCATTCGTTAAAGCCATGATCTTATCATCGTTATTAAAGAATGGGTTAGAACCAAAAGGTTTGCGTGTCAATGGTATTTCTTGTCGTGTGAGAAGCTCTT

novel_mir_231:

CTCGTTGTGCATTCGCCGAGACCCACAGTTGAGACCTCGGATGTCTCAGGTGTTGTGGATGTTGGAAAACGACGA

novel_mir_232:

ATCAAAATGTTCTGGATGGAGCTTTTGATGCTTTCGAGTCTCAAACATAAAAATCTAGTTTCTGTTATTGGGTTTTATGAATACAACACCGGGAAGATTATCTTATACAAGAGGGAGTCCAACGGAAGTCTCAAAACCTATCTAAGTGACAAGACCCT

novel_mir_233:

AGGGGTGCTATAAAGGTTTGGTTAATTCGAGTGTAAGGTTTTCTCTTAGGAATCCTGGTAAAAAAAGATGTTCTAATTTGTCCATTGCTTCTGTCCAGGGTATAAATTTCCGAGACGACTTTTGTTAGTGTTGAATCTAGGGTTCTTATTCCCGAGATGACTTTTGTTGGTGTCGAATCTAGAGTATCAAT

novel_mir_234:

TTTCGTGGCAGCTCGCCGTCGGCCGCCGTGACACCGCCCCCCCTCGCCGTCCAGCGCCGTTGGTTGAAGCTTCGTTCTGACGACA
