# Supplementary material for: MicroRNA Expression Profile during Aphid Feeding in Chrysanthemum (Chrysanthemum morifolium)
Source: PLoS One. 2015 Dec 9;10(12):e0143720. doi: 10.1371/journal.pone.0143720 (PMC4674109; doi:10.1371/journal.pone.0143720)
Supplement: S6 Table — (DOC) [file pone.0143720.s009.doc]

S6 Table. Stem-loop qRT-PCR primer.

| **miR-name** | **RT Primer** | **Forward Primer** | **Reverse Primer** |
| --- | --- | --- | --- |
| miR159a | gtcgtatccagtgcagggtccgaggtattcgcactggatacgacTAGAGC | agccgTTTGGATTGAAGGGA | agtgcagggtccgaggtat |
| miR160a | gtcgtatccagtgcagggtccgaggtattcgcactggatacgacTGGCAT | atgttTGCCTGGCTCCCTGT | agtgcagggtccgaggtat |
| miR169r-3p | gtcgtatccagtgcagggtccgaggtattcgcactggatacgacTTAGCC | tcgttGGCACGTGTCGTT | agtgcagggtccgaggtat |
| miR393a | gtcgtatccagtgcagggtccgaggtattcgcactggatacgacGGATCA | tgttTCCAAAGGGATCGCAT | agtgcagggtccgaggtat |
| miR408b | gtcgtatccagtgcagggtccgaggtattcgcactggatacgacAGGCCA | tattTGCACTGCCTCTTCCC | agtgcagggtccgaggtat |
| miR1511 | gtcgtatccagtgcagggtccgaggtattcgcactggatacgacTCTACA | agatgAACCTAGCTCTGATACCA | agtgcagggtccgaggtat |
| miR3449-3p | gtcgtatccagtgcagggtccgaggtattcgcactggatacgacTATGCC | gcccgAGATAGAAAGCTGATGT | agtgcagggtccgaggtat |
| miR5208d | gtcgtatccagtgcagggtccgaggtattcgcactggatacgacATGCCT | gcgggcATGATTAGTCATATCTTT | agtgcagggtccgaggtat |
| miR823 | gtcgtatccagtgcagggtccgaggtattcgcactggatacgacTACTTA | acggcTGGGTGGTGATTCAT | agtgcagggtccgaggtat |
| miR5200 | gtcgtatccagtgcagggtccgaggtattcgcactggatacgacAATCTT | acggcTGTAGATGACTCGCT | agtgcagggtccgaggtat |
